# Three crystal structures of the (4-bromo­benz­yl)tri­phenyl­phospho­nium cation with perchlorate, tetra­fluoro­borate and triiodide anions

**DOI:** 10.1107/S2056989026007292

**Published:** 2026-07-21

**Authors:** Will Lynch, Clifford Padgett, Kenadee Jones

**Affiliations:** a Department of Biochemistry Chemistry and Physics Center for Advanced Materials Science Georgia Southern University, 11935 Abercorn Street Savannah GA 31419 USA; b Department of Biochemistry Chemistry and Physics Georgia Southern University, 11935 Abercorn Street Savannah GA 31419 USA; University of Aberdeen, United Kingdom

**Keywords:** crystal structure, (4-bromo­benz­yl)tri­phenyl­phospho­nium ion, salts

## Abstract

The reaction of (4-bromo­benz­yl)tri­phenyl­phospho­nium bromide ([4BrBzTPP][Br]) with excess perchloric, tetra­fluoro­boric or hydro­iodic acid in methanol affords the corresponding anion-exchanged salts. X-ray quality crystals of (4-bromo­benz­yl)tri­phenyl­phospho­nium perchlorate, C_25_H_21_BrP^+^·ClO_4_^−^ or [4BrBzTPP][ClO_4_], (**I**), (4-bromo­benz­yl)tri­phenyl­phospho­nium tetra­fluoro­borate, C_25_H_21_BrP^+^·BF_4_^−^ or [4BrBzTPP][BF_4_], (**II**), and (4-bromo­benz­yl)tri­phenyl­phospho­nium triiodide, C_25_H_21_BrI_3_P^+^·I_3_^−^ or [4BrBzTPP][I_3_], (**III**), were isolated and characterized by single-crystal X-ray diffraction. Compounds (**I**) and (**II**) are isostructural and crystallize in the ortho­rhom­bic space group *Pbca*, whereas compound (**III**) crystallizes in the monoclinic space group *P*2_1_/*n*. In all three structures, the phospho­nium cation adopts a tetra­hedral geometry at phospho­rus, with C—P—C bond angles in the range 106.09 (11)–112.67 (11)°. Hirshfeld surface analyses show that H⋯H and H⋯C contacts dominate the environments of the (4-bromo­benz­yl)tri­phenyl­phospho­nium cations, whereas the perchlorate, tetra­fluoro­borate and triiodide anions are linked to the surrounding cations primarily through H⋯O, H⋯F and H⋯I contacts, respectively.

## Abstract 2

The crystal structures of three (4-bromo­benz­yl)tri­phenyl­phospho­nium salts with perchlorate, tetra­fluoro­borate and triiodide anions are reported and compared using Hirshfeld surface analysis.

## 1. Chemical context

There has been considerable recent inter­est in the use of benzyl­tri­phenyl­phospho­nium salts in optical applications. This has been a focal point of recent materials science research, especially for colorless salts of zinc(II) (Xu *et al.*, 2025 ▸) and manganese(II) (Yu *et al.*, 2024 ▸) containing 4-bromo­benzyl derivatives. Part of the focus of this research is the replacement of lead and cadmium based luminescent materials with less toxic alternatives, such as copper- and anti­mony-based materials (Fang *et al.*, 2021 ▸; Li *et al.*, 2022 ▸). Manganese-based phosphors have recently shown considerable promise in various photoelectric applications with ethyl­enebis(tri­phenyl­phospho­nium bromide) (Xu *et al.*, 2020 ▸). These manganese(II) metal halides were demonstrated to be green-emitting, with a photoluminescence quantum yield of up to 95%, and showed excellent X-ray scintillation properties. The manganese based derivatives using the title phospho­nium cation, 4-bromo­benzyl­tri­phenyl­phospho­nium (C_25_H_21_BrP^+^), were recently reported to have excellent quantum yields of over 96% and other optical characteristics attractive for medical imaging and radiation detection (Yu *et al.*, 2024 ▸). The corresponding tetra­bromo­zinc(II) complexes with 4-bromo­benzyl­tri­phenyl­phospho­nium also showed strong quantum yields of 87.6% and a stable color-rendering index of 88, indicating potential use in stable white-emitting LED applications (Xu *et al.*, 2025 ▸).
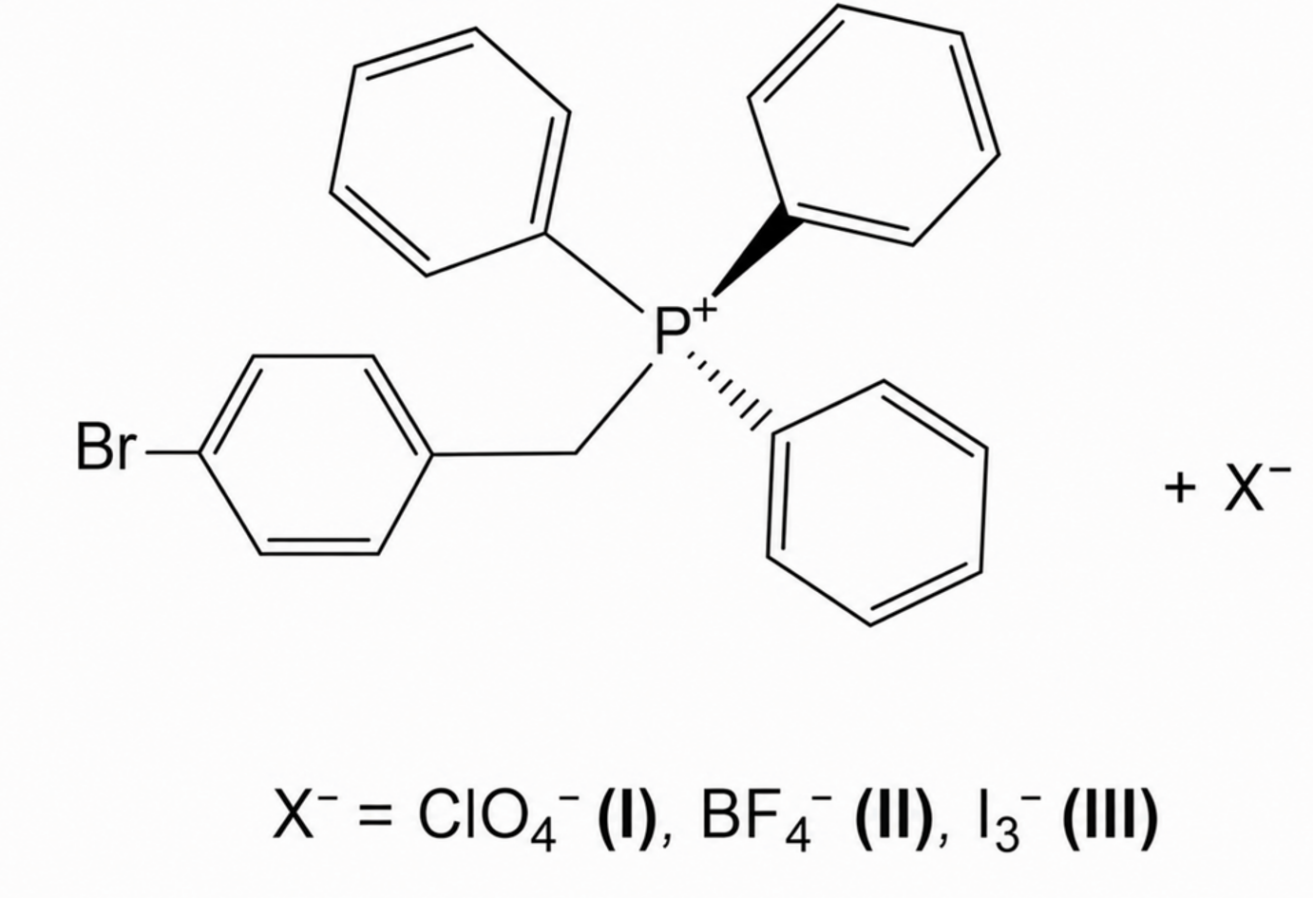


Due to the relatively small number of available crystal structures containing the 4-bromo­benzyl­tri­phenyl­phospho­nium cation, X-ray diffraction and IR spectroscopic studies were initiated to further investigate the solid-state structures of salts containing this cation with various anions. The synthesis is straightforward, involving replacement of the bromide anion in the starting material, 4-bromo­benzyl­tri­phenyl­phospho­nium bromide, by using an excess of the corresponding perchloric, tetra­fluoro­boric or hydro­iodic acid. The title salts are formulated as compound (**I**) ([4BrBzTPP][ClO_4_], C_25_H_21_BrClO_4_P), compound (**II**) ([4BrBzTPP][BF_4_], C_25_H_21_BBrF_4_P) and compound (**III**) ([4BrBzTPP][I_3_], C_25_H_21_BrI_3_P) and we now describe their structures.

## 2. Structural commentary

**Figure 1:**
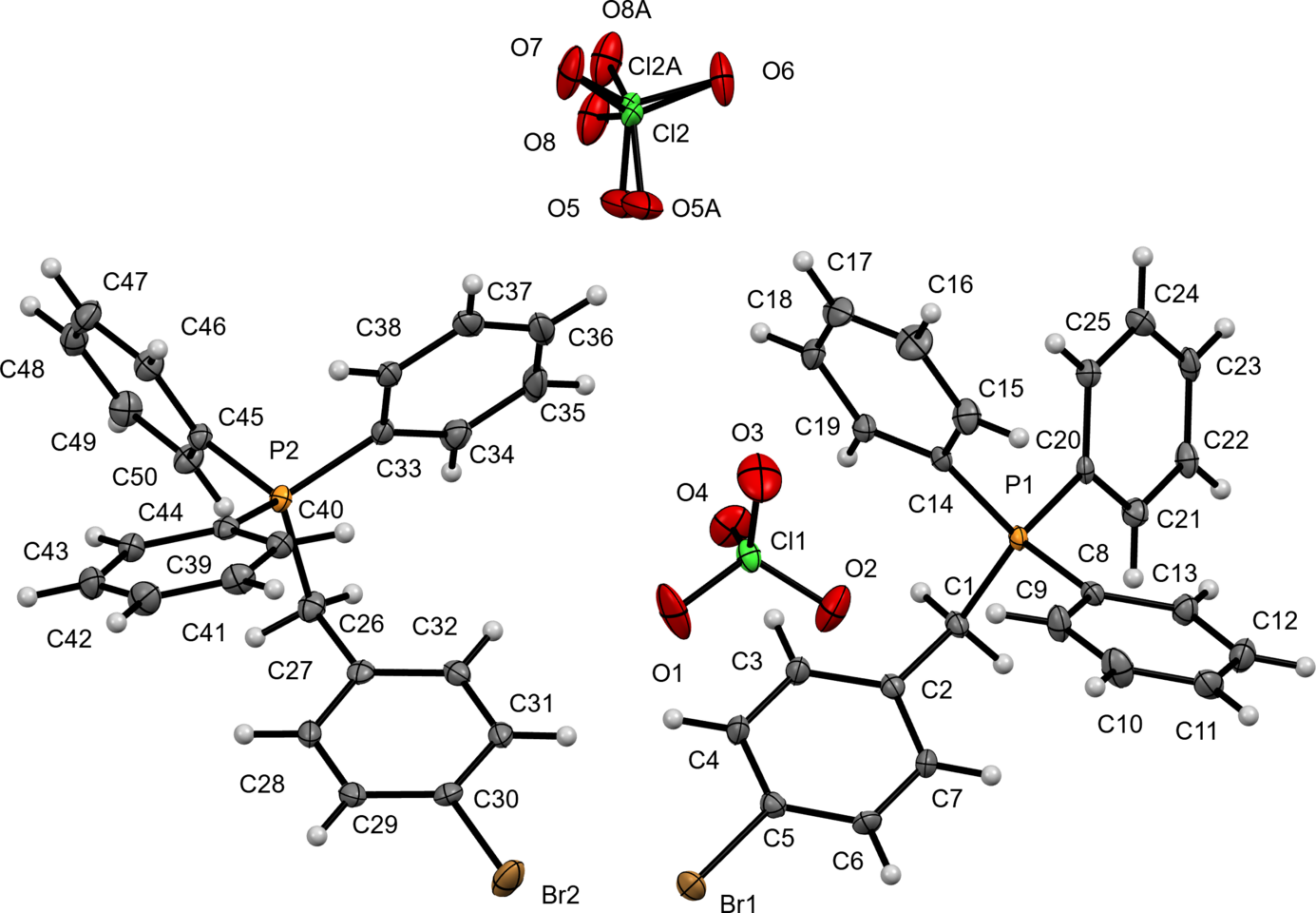
The mol­ecular structure of (**I**) with displacement ellipsoids drawn at the 50% probability level.

Compound (**I**) crystallizes in the centrosymmetric ortho­rhom­bic space group *Pbca*. The asymmetric unit consists of two formula units of the salt, [4BrBzTPP][ClO_4_] (Fig. 1 ▸). The phospho­rus atoms are observed to have slightly distorted tetra­hedral geometries, with C—P1—C bond angles in the range 106.09 (11)–112.37 (11)° and C—P2—C bond angles in the range 106.61 (11)–111.46 (11)°. The benzyl carbon atoms, C1 and C26, participate in angles that span most of these ranges, as determined by the solid-state packing. The perchlorate ion containing Cl1 is ordered, with O—Cl1—O bond angles in the range 108.38 (14)–110.44 (15)°. The second perchlorate ion was modeled as being positionally disordered over two orientations, with refined occupancies of 0.447 (3) and 0.553 (3) for the Cl2 and Cl2*A* components, respectively. The corresponding O—Cl—O angle ranges are 104.8 (5)–116.5 (5)° for the Cl2 component and 105.9 (4)–113.9 (4)° for the Cl2A component. The phenyl carbon-to-phospho­rus bond distances are in the ranges 1.789 (2)–1.797 (2) Å for P1 and 1.790 (2)–1.794 (2) Å for P2, whereas the benzyl carbon-to-phospho­rus bond distances are slightly longer, with C1—P1 = 1.812 (2) Å and C26—P2 = 1.811 (2) Å.

**Figure 2:**
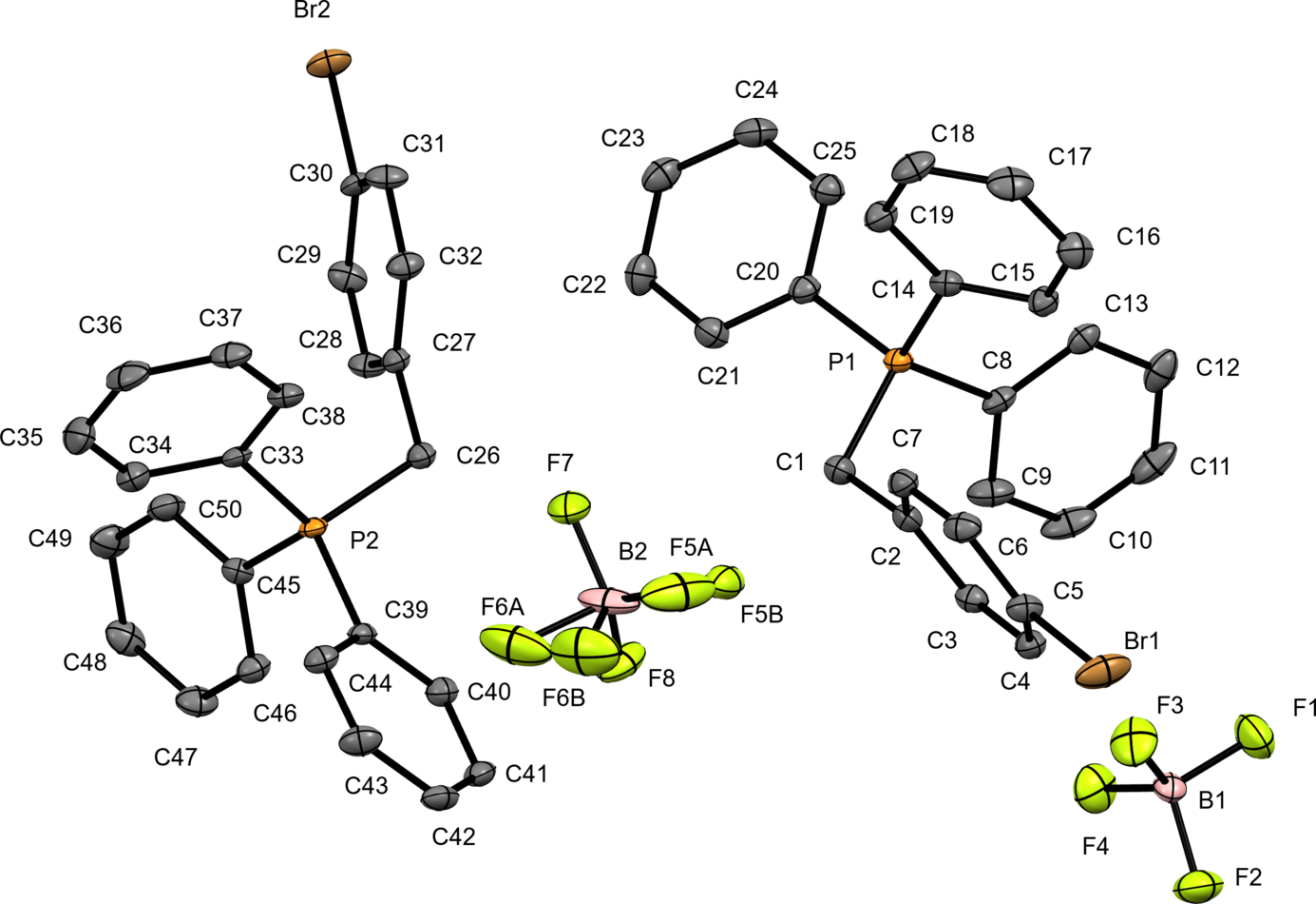
The mol­ecular structure of (**II**) with displacement ellipsoids drawn at the 50% probability level. Hydrogen atoms have been omitted for clarity.

Salt (**II**) also crystallizes in space group *Pbca*. The asymmetric unit consists of two formula units of the salt, [4BrBzTPP][BF_4_] (Fig. 2 ▸). The phospho­rus atoms have distorted tetra­hedral geometries, with C—P1—C bond angles in the range 106.48 (11)–111.42 (11)° and C—P2—C bond angles in the range 106.21 (11)–112.67 (11)°. The benzyl carbon atoms, C1 and C26, participate in angles within these ranges, and there is no clear distinction between the angles involving the benzyl carbon atoms and those involving the phenyl carbon atoms. The tetra­fluoro­borate anions also exhibit distorted tetra­hedral geometries. For the non-disordered BF_4_^−^ anion containing B1, the F—B1—F bond angles are in the range 107.9 (2)–110.3 (2)°. The B2 tetra­fluoro­borate anion is disordered, but the bond angles are generally consistent with a distorted tetra­hedral geometry. The phenyl carbon-to-phospho­rus bond distances are in the ranges 1.787 (2)–1.794 (2) Å for P1 and 1.790 (2)–1.799 (2) Å for P2. The benzyl carbon-to-phospho­rus bond distances are slightly longer, with C1—P1 = 1.816 (2) Å and C26—P2 = 1.816 (2) Å.

**Figure 3:**
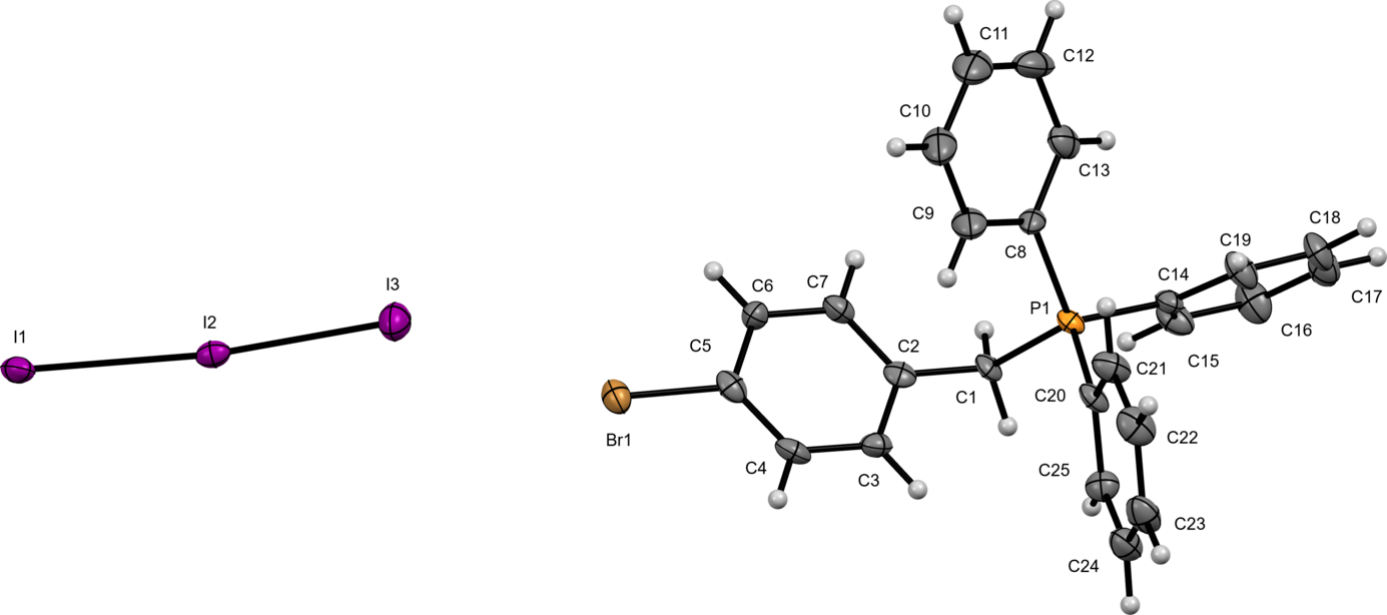
The mol­ecular structure of (**III**) with displacement ellipsoids drawn at the 50% probability level.

Salt (**III**) crystallizes in the centrosymmetric monoclinic space group *P*2_1_/*n*. The asymmetric unit consists of one formula unit of the salt, [4BrBzTPP][I_3_] (Fig. 3 ▸). The phospho­rus atom in the phospho­nium cation has an almost regular tetra­hedral geometry, with C—P1—C bond angles in the range 109.1 (2)–109.8 (2)°. This is a considerably narrower angular range than those observed in compounds (**I**) and (**II**). The triiodide anion is slightly bent, with an I3—I2—I1 bond angle of 173.489 (15)°. The I—I bond distances are unequal, as is commonly observed for I_3_^−^ anions, with I1—I2 = 3.0099 (5) Å and I2—I3 = 2.8603 (5) Å. The phenyl carbon-to-phospho­rus bond distances are in the range 1.790 (5)–1.800 (5) Å, whereas the benzyl carbon-to-phospho­rus bond distance is slightly longer, with C1—P1 = 1.818 (4) Å.

## 3. Supra­molecular features

**Figure 4:**
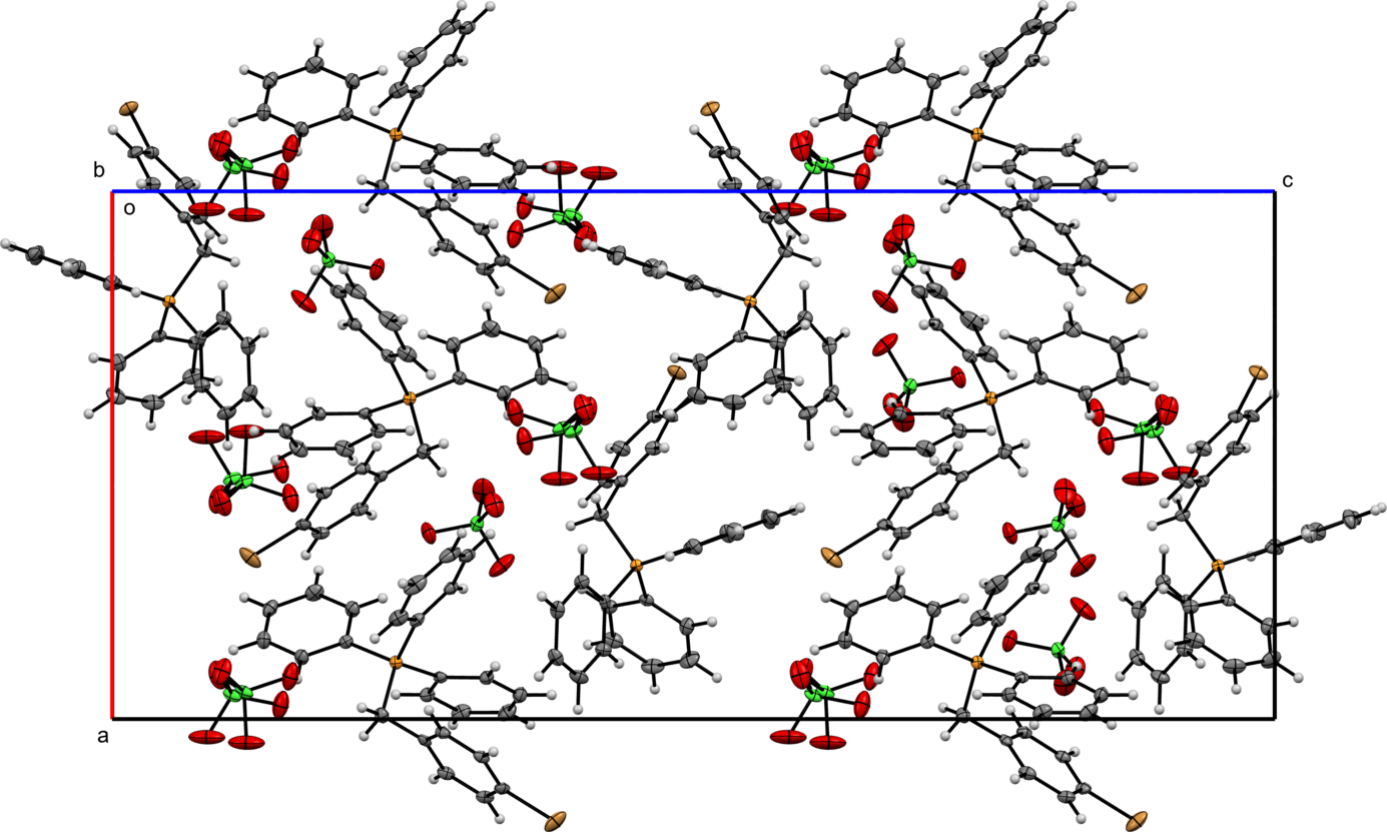
Crystal packing of compound (**I**), viewed down the *b-*axis direction.

Figs. 4 ▸, 5 ▸ and 6 ▸ illustrate the crystal packings of compounds (**I**), (**II**), and (**III**), respectively.

In compound (**I**), viewed down the *b-*axis direction (Fig. 4 ▸), the perchlorate anions occupy spaces between the phospho­nium cations and are incorporated into the packing through numerous C—H⋯O contacts (Table 1 ▸). The shortest C—H⋯O contacts include H21⋯O5*A* = 2.22 Å, H46⋯O4 = 2.31 Å, H1*A*⋯O2 = 2.35 Å and H26*A*⋯O2 = 2.36 Å. Additional Br⋯O contacts involving the bromo­benzyl substituent are present, including Br2⋯O6, Br2⋯O8A and Br2⋯O7 separations of 3.551 (2), 3.617 (5) and 3.730 (2) Å, respectively. The only significant π–π stacking inter­action involves the C27–C32 benzyl ring and the C33–C38 phenyl ring of an adjacent cation, related by the symmetry operation (−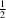 + *x*, *y*, 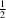 − *z*), with a centroid-to-centroid separation of 3.629 Å.

**Figure 5:**
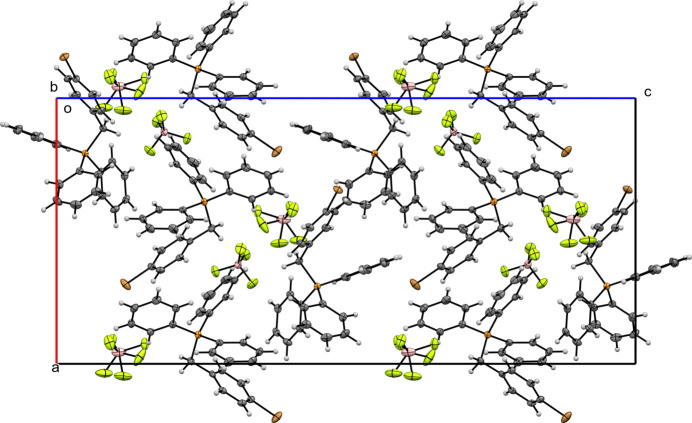
Crystal packing of compound (**II**), viewed down the *b*-axis direction.

In compound (**II**), viewed down the *b*-axis (Fig. 5 ▸), the tetra­fluoro­borate anions occupy spaces between the phospho­nium cations and are incorporated into the packing through numerous C—H⋯F contacts (Table 2 ▸). The shortest of these contacts include H40⋯F6B = 2.17 Å, H40⋯F6*A* = 2.25 Å, H25⋯F4 = 2.29 Å, H1*A*⋯F2 = 2.30 Å and H38⋯F1 = 2.37 Å. Additional close contacts involving the bromo­benzyl substituent are present, including Br1⋯Br2 and Br1⋯F8 separations of 3.9180 (4) and 3.496 (2) Å, respectively. The only significant π–π stacking inter­action involves the C2–C7 benzyl ring and the C8–C13 phenyl ring of an adjacent cation, generated by the symmetry operation (−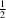 + *x*, *y*, 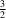 − *z*), with a centroid-to-centroid separation of 3.637 Å.

**Figure 6:**
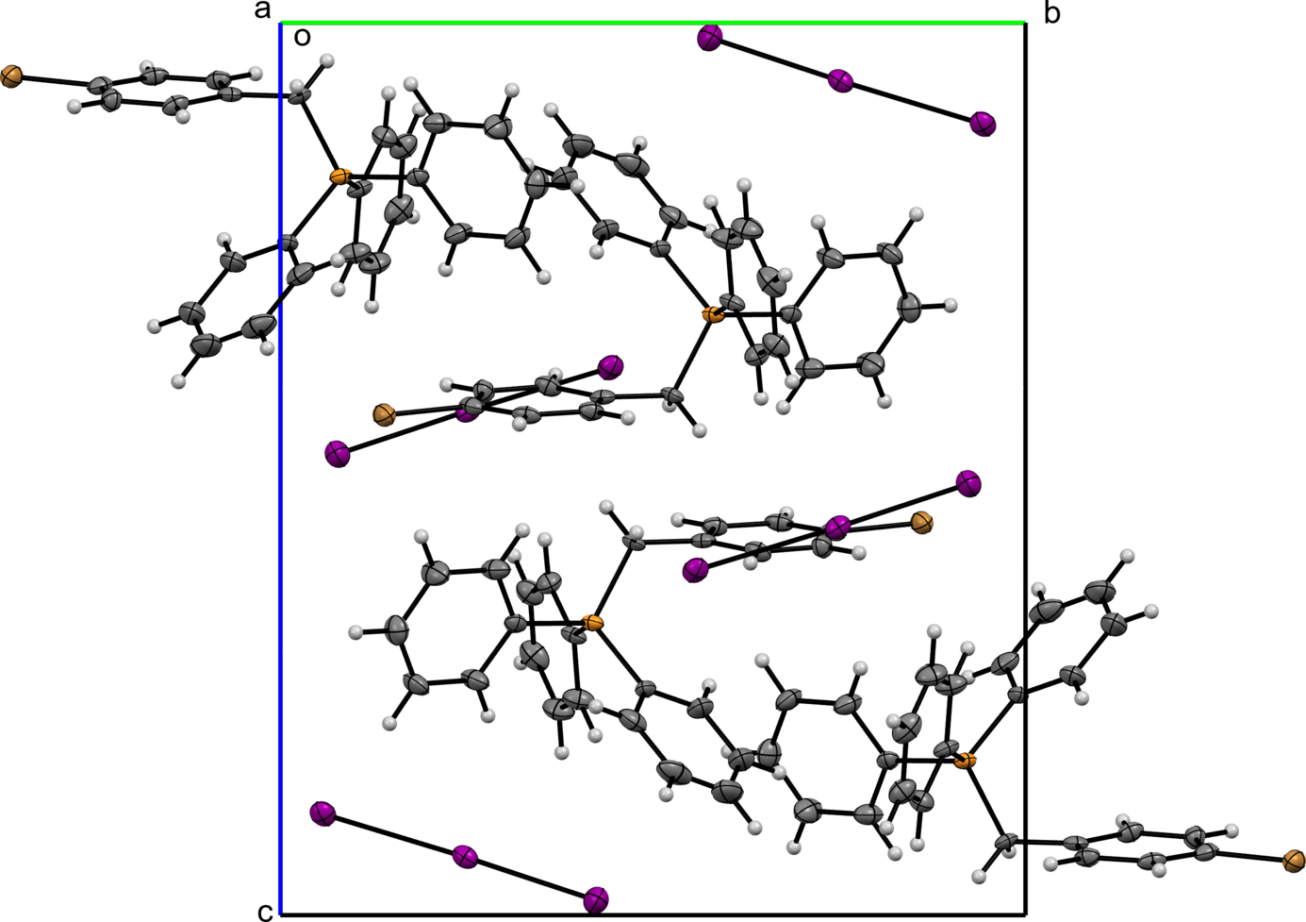
Crystal packing of compound (**III**), viewed down the *a*-axis direction.

In compound (**III**), viewed down the *a*-axis (Fig. 6 ▸), the triiodide anions are arranged between the phospho­nium cations. The packing (Table 3 ▸) is supported by H⋯I contacts involving the triiodide anion, with I1⋯H1b, I2⋯H7 and I1⋯H7 separations of 3.05, 3.06 and 3.11 Å, respectively. An I3⋯I3 contact of 3.9586 (8) Å is also present between neighboring triiodide anions. In addition, Br⋯I close contacts are observed, with Br1⋯I3 = 3.9333 (7) Å and Br1⋯I2 = 4.4364 (6) Å. No significant π–π stacking inter­actions are observed in this structure.

Hirshfeld surfaces were generated in *CrystalExplorer 21* (Spackman *et al.*, 2021 ▸) for each crystallographically independent (4-bromo­benz­yl)tri­phenyl­phospho­nium (4BrBzTPP) cation and for the corresponding anions, ClO_4_^−^ in (**I**), BF_4_^−^ in (**II**) and I_3_^−^ in (**III**). The corresponding two-dimensional fingerprint plots (McKinnon *et al.*, 2007 ▸) were analyzed to qu­antify the relative contributions of the various inter­molecular contacts. The results for (**I**), (**II**) and (**III**) are given in Tables 4 ▸–6 ▸ ▸, respectively.

For the 4BrBzTPP cations, the largest contributions to the Hirshfeld surfaces come from H⋯H and H⋯C/C⋯H contacts. These contacts account for 37.6–45.0% and 20.7–24.1%, respectively, of the cation surfaces in (**I**), 36.5–44.0% and 20.6–24.5% in (**II**), and 41.8% and 22.7% in (**III**). Contacts involving the anions make substantial additional contributions to the cation surfaces: H⋯O/O⋯H contacts contribute 14.9–20.8% in (**I**), H⋯F/F⋯H contacts contribute 15.7–21.6% in (**II**) and H⋯I/I⋯H contacts contribute 16.1% in (**III**). Contacts involving the bromine substituent also contribute to the cation surfaces, with H⋯Br/Br⋯H contacts ranging from 7.6 to 11.2%.

The anion Hirshfeld surfaces are dominated by contacts to hydrogen atoms of the surrounding cations. H⋯O/O⋯H contacts account for 92.5–95.0% of the perchlorate surfaces in (**I**), H⋯F/F⋯H contacts account for 92.9–96.4% of the tetra­fluoro­borate surfaces in (**II**) and H⋯I/I⋯H contacts account for 82.8% of the triiodide surface in (**III**). The triiodide surface also has contributions from C⋯I/I⋯C, Br⋯I/I⋯Br and I⋯I contacts of 9.4, 4.0 and 3.7%, respectively.

## 4. Database survey

A search of the web-based Cambridge Structural Database (CSD, accessed May 5, 2026; Groom *et al.*, 2016 ▸) for the (4-bromo­benz­yl)tri­phenyl­phospho­nium ion resulted in 23 unique entries. The entries most closely related to the present salts include tetra­halometallate salts, metal complex salts and simple halide/polyhalide derivatives. The tetra­halometallate salts include two tetra­bromido­manganate(II) salts, [MnBr_4_]^2–^ (COTZEU, Yu *et al.*, 2024 ▸; COTZEU01, Gong *et al.*, 2024 ▸), and one tetra­bromido­zincate(II) entry, [ZnBr_4_]^2−^ (EKEFOT; Xu *et al.*, 2025 ▸). Related Group 13 and Group 15 bromide salts have also been reported: [(4-bromo­benz­yl)tri­phenyl­phospho­nium] tetra­bromido­indium(III) (MUPVIG; Li & Wang, 2025*b* ▸) and [(4-bromo­benz­yl)tri­phenyl­phospho­nium] tetra­bromo­anti­mony(III) (MUPVEC; Li & Wang, 2025*a* ▸). Two isomeric copper iodide structures, bis­{[(4-bromo­phen­yl)meth­yl](triphen­yl)phosphanium} bis­(μ-iodido)­diiodido­dicopper(II), have also been reported (FUHYOA and FUHYOA01; Yang *et al.*, 2025 ▸).

Several sulfur-containing coordination-complex salts have also been reported, including bis­[(4-bromo­benz­yl)tri­phenyl­phospho­nium] bis­(1,2-di­cyano­ethane-1,2-di­thiol­ato-S,S′)nickel(II) monohydrate (DEGLOR; Yang & Ni, 2006 ▸), a (4-bromo­benz­yl)(triphen­yl)phospho­nium hemikis[tetra­kis­(iso­thio­cyanato)­cobaltate(II)] salt (KESHEX; Chen *et al.*, 2012 ▸), bis­[(4-bromo­benz­yl)tri­phenyl­phospho­nium] bis­(maleo­nitrile­dithiol­ato)copper(II) (PAKDEM; Chen *et al.*, 2011 ▸) and (4-bromo­benz­yl)tri­phenyl­phospho­nium bis­(maleo­nitrile­dithiol­ato)nickel(III) (PAKDAI; Chen *et al.*, 2011 ▸). Simple halide and polyhalide salts are also present, including (4-bromo­benz­yl)tri­phenyl­phospho­nium di­iodo­bromide (IXODUW; Chernov’yants *et al.*, 2016 ▸), bis­[(*p*-bromo­benz­yl)tri­phenyl­phospho­nium] tetra­bromide (TUMHOY; Vogt *et al.*, 1996 ▸), (p-bromo­benz­yl)tri­phenyl­phospho­nium tribromide di­chloro­methane solvate (TUMHUE; Vogt *et al.*, 1996 ▸) and (*p*-bromo­benz­yl)tri­phenyl­phospho­nium bromide di­chloro­methane solvate (TUMHIS; Vogt *et al.*, 1996 ▸).

## 5. Synthesis and crystallization

Compound (**I**) ([4BrBzTPP][ClO_4_]) was synthesized by dissolving 0.100 g of [4BrBzTPP][Br] (0.195 mmol, purchased from TCI America) in 5 ml of methanol, followed by the addition of 0.5 ml of HClO_4_ (70% in water, purchased from Sigma Millipore). The solution was stirred for 5 min, covered with a watch glass and allowed to stand. X-ray quality crystals were grown by slow evaporation at room temperature. Yield: 0.0542 g (52.2%). Selected IR bands (ATR-IR, cm^−1^): 3031 (*w*), 3008 (*w*), 2970 (*w*), 2861 (*w*), 2782 (*w*), 1488 (*m*), 1440 (*m*), 1104 (*m*), 722 (*s*), 679 (*s*), 530 (*s*), 512 (*s*), 490 (*s*).

Compound (**II**) ([4BrBzTPP][BF_4_]), was prepared by dissolving 0.100 g of [4BrBzTPP][Br] (0.195 mmol, purchased from TCI America) in 5 ml of methanol, followed by the addition of 0.5 ml of HBF_4_ (minimum 48% in water, purchased from Alfa Aesar) in one portion. The resulting solution was stirred for 5 min, covered with a watch glass and allowed to stand while the solvent slowly evaporated. X-ray quality crystals were grown by slow evaporation at room temperature. Yield: 0.0432 g (42.6%). Selected IR bands (ATR-IR, cm^−1^): 3040 (*w*), 3012 (*w*), 2968 (*w*), 2870 (*w*), 1478 (*m*), 1432 (*m*), 1099 (*m*), 730 (*s*), 684 (*s*), 532 (*s*), 510 (*s*), 498 (*s*).

Compound (**III**) ([4BrBzTPP][I_3_]) was synthesized by dissolving 0.100 g of [4BrBzTPP][Br] (0.195 mmol, purchased from TCI America) in 5 ml of methanol. Subsequently, 0.500 ml of HI (57% in water, purchased from Sigma Millipore) was added in one portion. The solution was covered with a watch glass and allowed to evaporate at room temperature. X-ray quality crystals were grown by slow evaporation at room temperature. After isolation, the sample had a mass of 0.0656 g (41.3%). Selected IR bands (ATR-IR, cm^−1^): 3038 (*w*), 3004 (*w*), 2974 (*w*), 2874 (*w*), 2760 (*w*), 1480 (*m*), 1436 (*m*), 1112 (*m*), 732 (*s*), 684 (*s*), 526 (*s*), 518 (*s*), 488 (*s*).

## 6. Refinement

Crystal data, data collection and structure refinement details are summarized in Table 7 ▸. H atoms were placed in calculated positions and refined as riding, with aromatic C—H = 0.95 Å and methyl­ene C—H = 0.99 Å, and with *U*_iso_(H) = 1.2*U*_eq_(C).

In compound (**I**), one perchlorate anion was modelled with positional disorder over two orientations, with refined occupancies of 0.447 (3) and 0.553 (3). The geometry of the disordered atoms was restrained using similar-distance restraints in *OLEX2*, and equivalent displacement parameters were applied to corresponding split atoms.

In compound (**II**), the tetra­fluoro­borate anion containing B2 was modelled as having positional disorder over two orientations, with refined occupancies of 0.587 (11) and 0.413 (11). The geometry of the disordered fluorine atoms was restrained using similar-distance restraints in *OLEX2*.

## Supplementary Material

Crystal structure: contains datablock(s) global, I, II, III. DOI: 10.1107/S2056989026007292/hb8239sup1.cif

Structure factors: contains datablock(s) I. DOI: 10.1107/S2056989026007292/hb8239Isup2.hkl

Structure factors: contains datablock(s) II. DOI: 10.1107/S2056989026007292/hb8239IIsup3.hkl

Structure factors: contains datablock(s) III. DOI: 10.1107/S2056989026007292/hb8239IIIsup4.hkl

Supporting information file. DOI: 10.1107/S2056989026007292/hb8239Isup5.cml

Supporting information file. DOI: 10.1107/S2056989026007292/hb8239IIsup6.cml

Supporting information file. DOI: 10.1107/S2056989026007292/hb8239IIIsup7.cml

CCDC references: 2573650, 2573649, 2573648

Additional supporting information:  crystallographic information; 3D view; checkCIF report

## Figures and Tables

**Table 1:** Hydrogen-bond geometry (Å, °) for (**I**)

*D*—H⋯*A*	*D*—H	H⋯*A*	*D*⋯*A*	*D*—H⋯*A*
C1—H1*A*⋯O2	0.99	2.35	3.223 (3)	146
C1—H1*B*⋯O5^i^	0.99	2.48	3.443 (6)	163
C12—H12⋯O6^ii^	0.95	2.58	3.297 (3)	133
C17—H17⋯O5	0.95	2.51	3.164 (6)	126
C19—H19⋯O3	0.95	2.45	3.347 (4)	156
C21—H21⋯O5*A*^i^	0.95	2.22	3.127 (5)	159
C21—H21⋯O5^i^	0.95	2.30	3.238 (5)	169
C23—H23⋯O1^i^	0.95	2.46	3.313 (3)	149
C26—H26*A*⋯O2^iii^	0.99	2.36	3.327 (3)	166
C26—H26*B*⋯O8*A*^iv^	0.99	2.50	3.424 (5)	154
C26—H26*B*⋯O8^iv^	0.99	2.52	3.388 (6)	146
C31—H31⋯O1	0.95	2.44	3.109 (3)	127
C42—H42⋯O6^v^	0.95	2.47	3.414 (3)	172
C46—H46⋯O4^v^	0.95	2.31	3.135 (3)	145

Symmetry codes: (i) 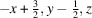; (ii) 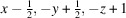; (iii) 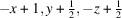; (iv) 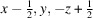; (v) 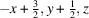.

**Table 2:** Hydrogen-bond geometry (Å, °) for (**II**)

*D*—H⋯*A*	*D*—H	H⋯*A*	*D*⋯*A*	*D*—H⋯*A*
C1—H1*A*⋯F2^i^	0.99	2.30	3.267 (3)	167
C1—H1*B*⋯F5*A*	0.99	2.52	3.355 (7)	142
C1—H1*B*⋯F5*B*	0.99	2.47	3.381 (6)	152
C4—H4⋯F3	0.95	2.47	3.112 (3)	125
C17—H17⋯F8^i^	0.95	2.40	3.344 (3)	175
C21—H21⋯F5*A*	0.95	2.54	3.443 (7)	159
C25—H25⋯F4^ii^	0.95	2.29	3.128 (3)	147
C26—H26*A*⋯F2^i^	0.99	2.41	3.255 (3)	143
C26—H26*B*⋯F6*A*	0.99	2.47	3.421 (4)	162
C38—H38⋯F1^i^	0.95	2.37	3.287 (3)	162
C40—H40⋯F6*A*	0.95	2.25	3.193 (4)	172
C40—H40⋯F6*B*	0.95	2.17	3.070 (6)	158
C42—H42⋯F3^iii^	0.95	2.42	3.287 (3)	151

Symmetry codes: (i) 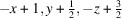; (ii) 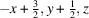; (iii) 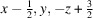.

**Table 3:** Hydrogen-bond geometry (Å, °) for (**III**)

*D*—H⋯*A*	*D*—H	H⋯*A*	*D*⋯*A*	*D*—H⋯*A*
C1—H1*B*⋯I1^i^	0.99	3.05	3.900 (4)	144
C7—H7⋯I2	0.95	3.06	3.716 (5)	128

Symmetry code: (i) 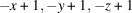.

**Table 4:** Contributions of selected inter­molecular contacts (%) to the Hirshfeld surfaces of the crystallographically independent ions in compound (**I**)

Contact	ClO_4_ 1	ClO_4_ 2	4BrBzTPP 1	4BrBzTPP 2
H⋯H	–	–	45.0	37.6
H⋯C	–	–	20.7	24.1
H⋯O	95.0	92.5	14.9	20.8
H⋯Br	–	–	10.7	7.7
C⋯O	4.9	0.0	0.0	1.6
C⋯C	–	–	2.1	5.1
C⋯Br	0.0	7.5	6.0	0.0
Br⋯Br	–	–	0.6	0.6
Br⋯O	–	–	0.0	2.5

**Table 5:** Contributions of selected inter­molecular contacts (%) to the Hirshfeld surfaces of the crystallographically independent ions in compound (**II**)

Contact	BF_4_ 1	BF_4_ 2	4BrBzTPP 1	4BrBzTPP 2
H⋯H	–	–	36.5	44.0
H⋯C	–	–	24.5	20.6
H⋯F	96.4	92.9	21.6	15.7
H⋯Br	–	–	7.6	10.5
C⋯F	3.5	0.0	1.3	0.1
C⋯C	–	–	5.2	2.3
C⋯Br	0.0	7.1	0.0	6.1
Br⋯Br	–	–	0.6	0.6

**Table 6:** Contributions of selected inter­molecular contacts (%) to the Hirshfeld surfaces of the crystallographically independent ions in compound (**III**)

Contact	I_3_	4BrBzTPP
H⋯H	–	41.8
H⋯C	–	22.7
H⋯Br	–	11.2
H⋯I	82.8	16.1
C⋯C	–	1.1
C⋯Br	–	3.0
C⋯I	9.4	2.9
I⋯Br	4.0	–
I⋯I	3.7	–

**Table 7:** Experimental details

	(**I**)	(**II**)	(**III**)
Crystal data			
Chemical formula	C_25_H_21_BrP^+^·ClO_4_^−^	C_25_H_21_BrP^+^·BF_4_^−^	C_25_H_21_BrP^+^·I_3_^−^
*M*_r_	531.75	519.11	813.00
Crystal system, space group	Orthorhombic, *Pbca*	Orthorhombic, *Pbca*	Monoclinic, *P*2_1_/*n*
Temperature (K)	110	110	111
*a*, *b*, *c* (Å)	15.2308 (1), 17.7829 (1), 33.5521 (2)	15.2560 (1), 17.6466 (1), 33.2354 (3)	9.8825 (1), 14.8840 (2), 17.8209 (2)
α, β, γ (°)	90, 90, 90	90, 90, 90	90, 91.504 (1), 90
*V* (Å^3^)	9087.51 (10)	8947.52 (11)	2620.39 (5)
*Z*	16	16	4
Radiation type	Cu *K*α	Cu *K*α	Cu *K*α
μ (mm^−1^)	4.47	3.57	30.54
Crystal size (mm)	0.38 × 0.28 × 0.22	0.38 × 0.28 × 0.22	0.12 × 0.10 × 0.04

Data collection			
Diffractometer	XtaLAB Synergy, Single source at home/near, HyPix-Bantam	XtaLAB Synergy, Single source at home/near, HyPix-Bantam	XtaLAB Synergy, Single source at home/near, HyPix-Bantam
Absorption correction	Multi-scan (*CrysAlis PRO* ; Rigaku OD, 2025 ▸)	Multi-scan (*CrysAlis PRO*; Rigaku OD, 2025 ▸)	Multi-scan (*CrysAlis PRO*; Rigaku OD, 2025 ▸)
*T*_min_, *T*_max_	0.793, 1.000	0.293, 1.000	0.233, 1.000
No. of measured, independent and observed [*I* > 2σ(*I*)] reflections	50580, 8471, 7550	33501, 8341, 7561	13993, 4888, 4462
*R*_int_	0.044	0.053	0.095
(sin θ/λ)_max_ (Å^−1^)	0.608	0.608	0.608

Refinement			
*R*[*F*^2^ > 2σ(*F*^2^)], *wR*(*F*^2^), *S*	0.037, 0.092, 1.03	0.041, 0.111, 1.01	0.044, 0.123, 1.03
No. of reflections	8471	8341	4888
No. of parameters	587	597	271
No. of restraints	9	2	0
H-atom treatment	H-atom parameters constrained	H-atom parameters constrained	H-atom parameters constrained
Δρ_max_, Δρ_min_ (e Å^−3^)	1.08, −1.11	0.91, −0.68	1.27, −1.71

Computer programs: *CrysAlis PRO* (Rigaku OD, 2025 ▸), *SHELXT* (Sheldrick, 2015*a* ▸), *SHELXL2018/3* (Sheldrick, 2015*b* ▸) and *OLEX2* (Dolomanov *et al.*, 2009 ▸).

## supplementary crystallographic information

### (4-Bromobenzyl)triphenylphosphonium perchlorate (I) Crystal data

**Table:**

C_25_H_21_BrP^+^·ClO_4_^−^	*D*_x_ = 1.555 Mg m^−^^3^
*M**_r_* = 531.75	Cu *K*α radiation, λ = 1.54184 Å
Orthorhombic, *P**b**c**a*	Cell parameters from 32962 reflections
*a* = 15.2308 (1) Å	θ = 2.5–69.4°
*b* = 17.7829 (1) Å	µ = 4.47 mm^−^^1^
*c* = 33.5521 (2) Å	*T* = 110 K
*V* = 9087.51 (10) Å^3^	Irregular, clear colourless
*Z* = 16	0.38 × 0.28 × 0.22 mm
*F*(000) = 4320	

### (4-Bromobenzyl)triphenylphosphonium perchlorate (I) Data collection

**Table:**

XtaLAB Synergy, Single source at home/near, HyPix-Bantam diffractometer	7550 reflections with *I* > 2σ(*I*)
Detector resolution: 10.0000 pixels mm^-1^	*R*_int_ = 0.044
ω scans	θ_max_ = 69.7°, θ_min_ = 2.6°
Absorption correction: multi-scan CrysAlisPro 1.171.44.128a (Rigaku OD, 2025)	*h* = −18→15
*T*_min_ = 0.793, *T*_max_ = 1.000	*k* = −18→21
50580 measured reflections	*l* = −40→39
8471 independent reflections	

### (4-Bromobenzyl)triphenylphosphonium perchlorate (I) Refinement

**Table:**

Refinement on *F*^2^	9 restraints
Least-squares matrix: full	Hydrogen site location: inferred from neighbouring sites
*R*[*F*^2^ > 2σ(*F*^2^)] = 0.037	H-atom parameters constrained
*wR*(*F*^2^) = 0.092	*w* = 1/[σ^2^(*F*_o_^2^) + (0.0431*P*)^2^ + 11.854*P*] where *P* = (*F*_o_^2^ + 2*F*_c_^2^)/3
*S* = 1.03	(Δ/σ)_max_ = 0.002
8471 reflections	Δρ_max_ = 1.08 e Å^−^^3^
587 parameters	Δρ_min_ = −1.10 e Å^−^^3^

### (4-Bromobenzyl)triphenylphosphonium perchlorate (I) Special details

**Table:**

Geometry. All esds (except the esd in the dihedral angle between two l.s. planes) are estimated using the full covariance matrix. The cell esds are taken into account individually in the estimation of esds in distances, angles and torsion angles; correlations between esds in cell parameters are only used when they are defined by crystal symmetry. An approximate (isotropic) treatment of cell esds is used for estimating esds involving l.s. planes.

### (4-Bromobenzyl)triphenylphosphonium perchlorate (I) Fractional atomic coordinates and isotropic or equivalent isotropic displacement parameters (Å^2^)

**Table:**

	*x*	*y*	*z*	*U*_iso_*/*U*_eq_	Occ. (<1)
Br1	0.34344 (2)	0.50713 (2)	0.48579 (2)	0.02288 (8)	
Br2	0.30592 (2)	0.59608 (2)	0.38115 (2)	0.03306 (9)	
Cl1	0.62998 (4)	0.39578 (3)	0.31367 (2)	0.01709 (13)	
P1	0.70910 (4)	0.25848 (3)	0.45110 (2)	0.01113 (12)	
P2	0.60715 (4)	0.75594 (3)	0.24417 (2)	0.01319 (13)	
Cl2	1.0448 (4)	0.6024 (4)	0.39682 (15)	0.0185 (6)	0.447 (3)
O4	0.64791 (14)	0.38820 (12)	0.27212 (6)	0.0324 (5)	
O6	1.09035 (15)	0.53537 (10)	0.40541 (6)	0.0350 (5)	
O7	1.08362 (16)	0.66735 (10)	0.40849 (6)	0.0383 (5)	
O2	0.59449 (15)	0.32613 (11)	0.32841 (6)	0.0366 (5)	
O1	0.56847 (16)	0.45514 (12)	0.31956 (7)	0.0449 (6)	
O3	0.70903 (16)	0.41188 (13)	0.33513 (7)	0.0477 (6)	
C2	0.54947 (15)	0.33465 (13)	0.43768 (7)	0.0143 (5)	
C31	0.42836 (15)	0.58221 (13)	0.31763 (7)	0.0168 (5)	
H31	0.437956	0.533131	0.327922	0.020*	
C20	0.77745 (15)	0.19408 (12)	0.42388 (7)	0.0120 (4)	
C39	0.57917 (15)	0.82238 (13)	0.28218 (7)	0.0139 (5)	
C1	0.61360 (15)	0.27811 (13)	0.42064 (7)	0.0148 (5)	
H1A	0.633910	0.296741	0.394406	0.018*	
H1B	0.581889	0.230310	0.415988	0.018*	
C32	0.47440 (16)	0.60709 (13)	0.28452 (7)	0.0166 (5)	
H32	0.516726	0.575148	0.272445	0.020*	
C40	0.57823 (16)	0.80006 (13)	0.32210 (7)	0.0168 (5)	
H40	0.599852	0.751956	0.329551	0.020*	
C27	0.45930 (15)	0.67848 (13)	0.26873 (7)	0.0150 (5)	
C45	0.64631 (15)	0.80570 (13)	0.20121 (7)	0.0146 (5)	
C44	0.54599 (16)	0.89232 (13)	0.27108 (8)	0.0182 (5)	
H44	0.546347	0.907386	0.243915	0.022*	
C4	0.48753 (16)	0.45963 (13)	0.43801 (8)	0.0192 (5)	
H4	0.487292	0.509826	0.428321	0.023*	
C29	0.35223 (15)	0.70124 (14)	0.32067 (8)	0.0188 (5)	
H29	0.310899	0.733375	0.333303	0.023*	
C38	0.74797 (15)	0.71264 (13)	0.29150 (7)	0.0169 (5)	
H38	0.746932	0.762413	0.301782	0.020*	
C30	0.36842 (15)	0.62969 (13)	0.33542 (7)	0.0168 (5)	
C28	0.39757 (15)	0.72483 (13)	0.28716 (8)	0.0183 (5)	
H28	0.386448	0.773423	0.276533	0.022*	
C3	0.54869 (16)	0.40827 (13)	0.42405 (7)	0.0176 (5)	
H3	0.590772	0.423762	0.404826	0.021*	
C8	0.67564 (15)	0.21714 (13)	0.49744 (7)	0.0143 (5)	
C13	0.67345 (16)	0.13886 (14)	0.50107 (8)	0.0187 (5)	
H13	0.691607	0.108042	0.479460	0.022*	
C14	0.77460 (15)	0.34045 (12)	0.45960 (7)	0.0140 (5)	
C25	0.86838 (16)	0.20457 (13)	0.42252 (7)	0.0173 (5)	
H25	0.894563	0.245909	0.435952	0.021*	
C50	0.61725 (17)	0.78751 (14)	0.16296 (7)	0.0190 (5)	
H50	0.575651	0.748346	0.159175	0.023*	
C5	0.42699 (15)	0.43697 (13)	0.46614 (7)	0.0158 (5)	
C19	0.78734 (16)	0.38965 (13)	0.42758 (8)	0.0169 (5)	
H19	0.753333	0.384793	0.403983	0.020*	
C26	0.50712 (16)	0.70586 (14)	0.23191 (7)	0.0185 (5)	
H26A	0.467900	0.739506	0.216559	0.022*	
H26B	0.521672	0.662238	0.214811	0.022*	
C33	0.68750 (16)	0.69091 (13)	0.26241 (7)	0.0158 (5)	
C15	0.82456 (17)	0.34769 (14)	0.49424 (8)	0.0211 (5)	
H15	0.815732	0.314291	0.515969	0.025*	
C23	0.88305 (17)	0.09403 (13)	0.38159 (7)	0.0192 (5)	
H23	0.919169	0.060208	0.367039	0.023*	
C22	0.79310 (17)	0.08318 (13)	0.38300 (7)	0.0187 (5)	
H22	0.767547	0.041530	0.369584	0.022*	
C41	0.54555 (17)	0.84860 (14)	0.35069 (8)	0.0212 (5)	
H41	0.545831	0.834210	0.377953	0.025*	
C21	0.74009 (16)	0.13290 (13)	0.40393 (7)	0.0176 (5)	
H21	0.678363	0.125354	0.404706	0.021*	
C7	0.48691 (15)	0.31279 (13)	0.46567 (7)	0.0179 (5)	
H7	0.486176	0.262348	0.474978	0.022*	
C48	0.70942 (17)	0.88458 (14)	0.13599 (8)	0.0221 (5)	
H48	0.730219	0.912264	0.113688	0.027*	
C18	0.85060 (17)	0.44582 (14)	0.43082 (9)	0.0225 (6)	
H18	0.859648	0.479705	0.409319	0.027*	
C6	0.42577 (16)	0.36362 (14)	0.48010 (7)	0.0191 (5)	
H6	0.383549	0.348404	0.499320	0.023*	
C49	0.64970 (17)	0.82716 (15)	0.13047 (8)	0.0224 (5)	
H49	0.630675	0.814693	0.104326	0.027*	
C24	0.92005 (17)	0.15425 (14)	0.40143 (8)	0.0229 (6)	
H24	0.981854	0.161282	0.400613	0.027*	
C46	0.70824 (17)	0.86281 (14)	0.20666 (8)	0.0201 (5)	
H46	0.728862	0.874566	0.232651	0.024*	
C43	0.51234 (17)	0.93983 (14)	0.30025 (8)	0.0233 (6)	
H43	0.489191	0.987484	0.292951	0.028*	
C12	0.64475 (18)	0.10634 (14)	0.53623 (8)	0.0239 (6)	
H12	0.642838	0.053137	0.538646	0.029*	
C42	0.51241 (18)	0.91811 (15)	0.33973 (8)	0.0258 (6)	
H42	0.489601	0.951000	0.359488	0.031*	
C47	0.73929 (18)	0.90209 (14)	0.17400 (8)	0.0245 (6)	
H47	0.781139	0.941133	0.177566	0.029*	
C37	0.80938 (17)	0.66163 (15)	0.30534 (8)	0.0238 (6)	
H37	0.850380	0.676445	0.325181	0.029*	
C11	0.61895 (17)	0.15087 (15)	0.56774 (8)	0.0231 (6)	
H11	0.599320	0.128224	0.591785	0.028*	
C17	0.90017 (18)	0.45263 (14)	0.46497 (9)	0.0264 (6)	
H17	0.943465	0.490941	0.466732	0.032*	
C9	0.64872 (17)	0.26207 (14)	0.52937 (8)	0.0217 (5)	
H9	0.649093	0.315297	0.526960	0.026*	
C10	0.62151 (19)	0.22857 (15)	0.56452 (8)	0.0261 (6)	
H10	0.604546	0.258938	0.586505	0.031*	
C34	0.69172 (17)	0.61828 (14)	0.24639 (9)	0.0239 (6)	
H34	0.652349	0.603542	0.225861	0.029*	
C16	0.88754 (19)	0.40434 (15)	0.49666 (9)	0.0283 (6)	
H16	0.921802	0.409735	0.520149	0.034*	
C35	0.75378 (19)	0.56803 (14)	0.26070 (9)	0.0310 (7)	
H35	0.756790	0.518630	0.250010	0.037*	
C36	0.81135 (19)	0.58953 (16)	0.29050 (9)	0.0303 (7)	
H36	0.852447	0.554326	0.300764	0.036*	
O8	1.0268 (4)	0.6102 (3)	0.35497 (17)	0.0394 (10)	0.447 (3)
O5	0.9658 (3)	0.5969 (3)	0.4185 (2)	0.0571 (12)	0.447 (3)
O5A	0.9554 (3)	0.5938 (3)	0.38440 (19)	0.0571 (12)	0.553 (3)
Cl2A	1.0516 (3)	0.5996 (3)	0.38644 (12)	0.0185 (6)	0.553 (3)
O8A	1.0810 (4)	0.6004 (2)	0.34660 (12)	0.0394 (10)	0.553 (3)

### (4-Bromobenzyl)triphenylphosphonium perchlorate (I) Atomic displacement parameters (Å^2^)

**Table:**

	*U*^11^	*U*^22^	*U*^33^	*U*^12^	*U*^13^	*U*^23^
Br1	0.02016 (14)	0.01737 (13)	0.03113 (16)	0.00512 (11)	0.00817 (11)	−0.00313 (11)
Br2	0.03696 (17)	0.02090 (15)	0.04132 (19)	−0.00296 (13)	0.02256 (14)	0.00377 (12)
Cl1	0.0219 (3)	0.0126 (3)	0.0168 (3)	0.0035 (2)	−0.0023 (2)	−0.0036 (2)
P1	0.0127 (3)	0.0072 (3)	0.0135 (3)	0.0016 (2)	0.0000 (2)	−0.0004 (2)
P2	0.0157 (3)	0.0100 (3)	0.0138 (3)	−0.0013 (2)	−0.0004 (2)	−0.0007 (2)
Cl2	0.0158 (8)	0.0121 (4)	0.028 (2)	−0.0001 (5)	0.0070 (12)	0.0064 (12)
O4	0.0427 (12)	0.0358 (11)	0.0186 (9)	−0.0026 (10)	0.0098 (9)	−0.0051 (9)
O6	0.0596 (14)	0.0139 (9)	0.0314 (11)	0.0093 (10)	−0.0134 (10)	0.0012 (8)
O7	0.0700 (16)	0.0119 (9)	0.0331 (11)	−0.0042 (10)	0.0115 (11)	−0.0022 (8)
O2	0.0544 (13)	0.0205 (9)	0.0349 (11)	−0.0107 (10)	0.0078 (10)	0.0018 (9)
O1	0.0592 (15)	0.0328 (11)	0.0427 (13)	0.0326 (11)	0.0048 (12)	−0.0049 (10)
O3	0.0458 (13)	0.0409 (13)	0.0565 (15)	−0.0057 (11)	−0.0334 (12)	−0.0027 (11)
C2	0.0139 (11)	0.0130 (11)	0.0160 (11)	0.0014 (10)	−0.0020 (9)	−0.0010 (9)
C31	0.0144 (11)	0.0132 (11)	0.0228 (13)	−0.0011 (10)	−0.0043 (10)	0.0015 (10)
C20	0.0171 (11)	0.0078 (10)	0.0112 (10)	0.0029 (9)	0.0010 (9)	0.0024 (9)
C39	0.0117 (10)	0.0131 (11)	0.0167 (11)	−0.0023 (9)	−0.0008 (9)	−0.0036 (9)
C1	0.0144 (11)	0.0108 (10)	0.0191 (12)	0.0023 (9)	−0.0023 (10)	−0.0016 (9)
C32	0.0135 (11)	0.0156 (11)	0.0208 (12)	0.0016 (10)	−0.0018 (10)	−0.0039 (10)
C40	0.0169 (11)	0.0158 (11)	0.0176 (12)	−0.0005 (10)	0.0006 (10)	0.0008 (10)
C27	0.0124 (10)	0.0155 (11)	0.0172 (12)	−0.0038 (10)	−0.0048 (9)	0.0001 (9)
C45	0.0155 (11)	0.0116 (10)	0.0167 (11)	−0.0004 (10)	0.0012 (9)	−0.0008 (9)
C44	0.0175 (11)	0.0162 (11)	0.0208 (12)	0.0004 (10)	0.0011 (10)	0.0033 (10)
C4	0.0202 (12)	0.0108 (11)	0.0266 (13)	0.0010 (10)	0.0053 (11)	0.0031 (10)
C29	0.0129 (11)	0.0159 (12)	0.0275 (13)	0.0002 (10)	−0.0014 (10)	−0.0007 (10)
C38	0.0160 (11)	0.0162 (11)	0.0186 (12)	0.0009 (10)	0.0044 (10)	0.0050 (10)
C30	0.0122 (10)	0.0173 (11)	0.0210 (12)	−0.0040 (10)	0.0004 (10)	0.0013 (10)
C28	0.0158 (11)	0.0126 (11)	0.0266 (13)	−0.0003 (10)	−0.0040 (10)	0.0039 (10)
C3	0.0188 (12)	0.0129 (11)	0.0210 (12)	0.0008 (10)	0.0047 (10)	0.0024 (10)
C8	0.0131 (10)	0.0134 (11)	0.0163 (11)	0.0036 (10)	0.0010 (9)	0.0010 (9)
C13	0.0218 (12)	0.0137 (11)	0.0205 (12)	0.0009 (10)	0.0041 (10)	−0.0010 (10)
C14	0.0136 (11)	0.0098 (10)	0.0186 (11)	0.0030 (9)	0.0021 (9)	−0.0017 (9)
C25	0.0167 (11)	0.0128 (11)	0.0223 (12)	0.0009 (10)	0.0007 (10)	0.0005 (10)
C50	0.0213 (12)	0.0179 (12)	0.0179 (12)	−0.0041 (11)	−0.0004 (10)	−0.0029 (10)
C5	0.0142 (11)	0.0142 (11)	0.0189 (12)	0.0019 (10)	0.0007 (10)	−0.0018 (10)
C19	0.0167 (11)	0.0117 (11)	0.0223 (13)	0.0010 (10)	−0.0005 (10)	−0.0005 (10)
C26	0.0208 (12)	0.0164 (11)	0.0182 (12)	−0.0027 (10)	−0.0019 (10)	−0.0009 (10)
C33	0.0171 (11)	0.0112 (11)	0.0191 (12)	−0.0002 (10)	0.0053 (10)	0.0016 (9)
C15	0.0266 (13)	0.0164 (12)	0.0201 (13)	0.0015 (11)	−0.0020 (11)	−0.0026 (10)
C23	0.0252 (13)	0.0124 (11)	0.0201 (12)	0.0081 (10)	0.0065 (11)	0.0021 (10)
C22	0.0275 (13)	0.0107 (11)	0.0178 (12)	0.0043 (10)	−0.0011 (10)	−0.0026 (9)
C41	0.0228 (12)	0.0233 (13)	0.0176 (12)	−0.0009 (11)	0.0025 (10)	−0.0021 (10)
C21	0.0181 (12)	0.0148 (11)	0.0200 (12)	0.0005 (10)	−0.0013 (10)	−0.0018 (10)
C7	0.0169 (11)	0.0117 (11)	0.0252 (13)	0.0007 (10)	0.0001 (10)	0.0056 (10)
C48	0.0266 (13)	0.0183 (12)	0.0216 (13)	0.0031 (11)	0.0072 (11)	0.0041 (11)
C18	0.0222 (13)	0.0105 (11)	0.0347 (15)	−0.0004 (10)	0.0050 (12)	0.0014 (11)
C6	0.0141 (11)	0.0216 (12)	0.0215 (13)	0.0000 (10)	0.0061 (10)	0.0062 (10)
C49	0.0270 (13)	0.0238 (13)	0.0163 (12)	0.0006 (11)	0.0005 (11)	−0.0004 (11)
C24	0.0174 (12)	0.0197 (12)	0.0317 (14)	0.0036 (11)	0.0060 (11)	0.0008 (11)
C46	0.0224 (12)	0.0185 (12)	0.0196 (12)	−0.0021 (11)	−0.0003 (10)	−0.0032 (10)
C43	0.0237 (13)	0.0133 (11)	0.0330 (15)	0.0020 (11)	0.0053 (12)	−0.0004 (11)
C12	0.0272 (13)	0.0163 (12)	0.0281 (14)	−0.0005 (11)	0.0036 (12)	0.0054 (11)
C42	0.0274 (14)	0.0196 (12)	0.0304 (15)	0.0013 (12)	0.0096 (12)	−0.0062 (11)
C47	0.0267 (14)	0.0166 (12)	0.0300 (14)	−0.0060 (11)	0.0042 (12)	−0.0003 (11)
C37	0.0174 (12)	0.0288 (14)	0.0252 (14)	0.0028 (11)	0.0070 (11)	0.0106 (12)
C11	0.0202 (12)	0.0269 (13)	0.0221 (13)	0.0054 (11)	0.0059 (11)	0.0083 (11)
C17	0.0198 (12)	0.0131 (11)	0.0463 (17)	−0.0034 (11)	−0.0007 (12)	−0.0085 (12)
C9	0.0292 (13)	0.0144 (12)	0.0213 (13)	0.0072 (11)	0.0040 (11)	0.0012 (10)
C10	0.0332 (15)	0.0248 (13)	0.0203 (13)	0.0097 (12)	0.0067 (12)	−0.0015 (11)
C34	0.0235 (13)	0.0160 (12)	0.0323 (15)	−0.0022 (11)	0.0070 (12)	−0.0037 (11)
C16	0.0273 (14)	0.0233 (13)	0.0343 (16)	−0.0049 (12)	−0.0089 (13)	−0.0097 (12)
C35	0.0344 (15)	0.0120 (12)	0.0466 (18)	0.0057 (12)	0.0176 (15)	0.0037 (12)
C36	0.0249 (14)	0.0261 (14)	0.0401 (17)	0.0111 (12)	0.0143 (13)	0.0171 (13)
O8	0.068 (3)	0.0253 (15)	0.0252 (18)	−0.010 (2)	−0.011 (2)	0.0055 (13)
O5	0.0165 (14)	0.0387 (17)	0.116 (4)	0.0021 (13)	0.002 (2)	0.008 (3)
O5A	0.0165 (14)	0.0387 (17)	0.116 (4)	0.0021 (13)	0.002 (2)	0.008 (3)
Cl2A	0.0158 (8)	0.0121 (4)	0.028 (2)	−0.0001 (5)	0.0070 (12)	0.0064 (12)
O8A	0.068 (3)	0.0253 (15)	0.0252 (18)	−0.010 (2)	−0.011 (2)	0.0055 (13)

### (4-Bromobenzyl)triphenylphosphonium perchlorate (I) Geometric parameters (Å, º)

**Table:**

Br1—C5	1.900 (2)	C14—C19	1.399 (3)
Br2—C30	1.902 (2)	C14—C15	1.395 (3)
Cl1—O4	1.4270 (19)	C25—H25	0.9500
Cl1—O2	1.4390 (19)	C25—C24	1.386 (3)
Cl1—O1	1.425 (2)	C50—H50	0.9500
Cl1—O3	1.432 (2)	C50—C49	1.389 (4)
P1—C20	1.797 (2)	C5—C6	1.386 (3)
P1—C1	1.812 (2)	C19—H19	0.9500
P1—C8	1.794 (2)	C19—C18	1.392 (3)
P1—C14	1.789 (2)	C26—H26A	0.9900
P2—C39	1.790 (2)	C26—H26B	0.9900
P2—C45	1.794 (2)	C33—C34	1.401 (3)
P2—C26	1.812 (2)	C15—H15	0.9500
P2—C33	1.791 (2)	C15—C16	1.393 (4)
Cl2—O6	1.409 (7)	C23—H23	0.9500
Cl2—O7	1.356 (7)	C23—C22	1.384 (4)
Cl2—O8	1.437 (6)	C23—C24	1.381 (4)
Cl2—O5	1.409 (7)	C22—H22	0.9500
O6—Cl2A	1.435 (6)	C22—C21	1.388 (3)
O7—Cl2A	1.496 (5)	C41—H41	0.9500
C2—C1	1.514 (3)	C41—C42	1.385 (4)
C2—C3	1.387 (3)	C21—H21	0.9500
C2—C7	1.393 (3)	C7—H7	0.9500
C31—H31	0.9500	C7—C6	1.385 (3)
C31—C32	1.386 (3)	C48—H48	0.9500
C31—C30	1.379 (3)	C48—C49	1.380 (4)
C20—C25	1.398 (3)	C48—C47	1.390 (4)
C20—C21	1.398 (3)	C18—H18	0.9500
C39—C40	1.397 (3)	C18—C17	1.378 (4)
C39—C44	1.393 (3)	C6—H6	0.9500
C1—H1A	0.9900	C49—H49	0.9500
C1—H1B	0.9900	C24—H24	0.9500
C32—H32	0.9500	C46—H46	0.9500
C32—C27	1.395 (3)	C46—C47	1.383 (4)
C40—H40	0.9500	C43—H43	0.9500
C40—C41	1.383 (3)	C43—C42	1.380 (4)
C27—C28	1.395 (3)	C12—H12	0.9500
C27—C26	1.514 (3)	C12—C11	1.378 (4)
C45—C50	1.395 (3)	C42—H42	0.9500
C45—C46	1.398 (3)	C47—H47	0.9500
C44—H44	0.9500	C37—H37	0.9500
C44—C43	1.391 (4)	C37—C36	1.376 (4)
C4—H4	0.9500	C11—H11	0.9500
C4—C3	1.386 (3)	C11—C10	1.387 (4)
C4—C5	1.380 (3)	C17—H17	0.9500
C29—H29	0.9500	C17—C16	1.380 (4)
C29—C30	1.387 (3)	C9—H9	0.9500
C29—C28	1.385 (4)	C9—C10	1.385 (4)
C38—H38	0.9500	C10—H10	0.9500
C38—C33	1.397 (3)	C34—H34	0.9500
C38—C37	1.383 (3)	C34—C35	1.387 (4)
C28—H28	0.9500	C16—H16	0.9500
C3—H3	0.9500	C35—H35	0.9500
C8—C13	1.398 (3)	C35—C36	1.384 (4)
C8—C9	1.398 (3)	C36—H36	0.9500
C13—H13	0.9500	O5A—Cl2A	1.470 (6)
C13—C12	1.385 (4)	Cl2A—O8A	1.410 (5)
			
O4—Cl1—O2	109.05 (12)	C14—C19—H19	120.5
O4—Cl1—O3	110.44 (15)	C18—C19—C14	119.0 (2)
O1—Cl1—O4	109.35 (13)	C18—C19—H19	120.5
O1—Cl1—O2	110.05 (14)	P2—C26—H26A	109.2
O1—Cl1—O3	109.57 (14)	P2—C26—H26B	109.2
O3—Cl1—O2	108.38 (14)	C27—C26—P2	112.16 (16)
C20—P1—C1	107.52 (11)	C27—C26—H26A	109.2
C8—P1—C20	110.12 (10)	C27—C26—H26B	109.2
C8—P1—C1	109.86 (11)	H26A—C26—H26B	107.9
C14—P1—C20	106.09 (11)	C38—C33—P2	120.71 (18)
C14—P1—C1	112.37 (11)	C38—C33—C34	119.6 (2)
C14—P1—C8	110.74 (11)	C34—C33—P2	119.7 (2)
C39—P2—C45	109.03 (11)	C14—C15—H15	120.3
C39—P2—C26	106.61 (11)	C16—C15—C14	119.4 (2)
C39—P2—C33	110.21 (11)	C16—C15—H15	120.3
C45—P2—C26	109.85 (11)	C22—C23—H23	120.2
C33—P2—C45	111.46 (11)	C24—C23—H23	120.2
C33—P2—C26	109.55 (11)	C24—C23—C22	119.7 (2)
O6—Cl2—O8	112.1 (4)	C23—C22—H22	119.9
O6—Cl2—O5	104.8 (5)	C23—C22—C21	120.3 (2)
O7—Cl2—O6	116.5 (5)	C21—C22—H22	119.9
O7—Cl2—O8	106.4 (5)	C40—C41—H41	119.9
O7—Cl2—O5	106.4 (4)	C40—C41—C42	120.3 (2)
O5—Cl2—O8	110.3 (6)	C42—C41—H41	119.9
C3—C2—C1	120.5 (2)	C20—C21—H21	120.0
C3—C2—C7	118.7 (2)	C22—C21—C20	120.1 (2)
C7—C2—C1	120.7 (2)	C22—C21—H21	120.0
C32—C31—H31	120.5	C2—C7—H7	119.6
C30—C31—H31	120.5	C6—C7—C2	120.9 (2)
C30—C31—C32	119.1 (2)	C6—C7—H7	119.6
C25—C20—P1	120.36 (18)	C49—C48—H48	119.8
C25—C20—C21	119.4 (2)	C49—C48—C47	120.3 (2)
C21—C20—P1	120.22 (18)	C47—C48—H48	119.8
C40—C39—P2	119.87 (18)	C19—C18—H18	119.8
C44—C39—P2	119.03 (18)	C17—C18—C19	120.5 (2)
C44—C39—C40	120.4 (2)	C17—C18—H18	119.8
P1—C1—H1A	108.4	C5—C6—H6	120.4
P1—C1—H1B	108.4	C7—C6—C5	119.1 (2)
C2—C1—P1	115.65 (16)	C7—C6—H6	120.4
C2—C1—H1A	108.4	C50—C49—H49	119.8
C2—C1—H1B	108.4	C48—C49—C50	120.3 (2)
H1A—C1—H1B	107.4	C48—C49—H49	119.8
C31—C32—H32	119.6	C25—C24—H24	119.5
C31—C32—C27	120.8 (2)	C23—C24—C25	121.0 (2)
C27—C32—H32	119.6	C23—C24—H24	119.5
C39—C40—H40	120.3	C45—C46—H46	120.2
C41—C40—C39	119.4 (2)	C47—C46—C45	119.6 (2)
C41—C40—H40	120.3	C47—C46—H46	120.2
C32—C27—C28	118.7 (2)	C44—C43—H43	119.8
C32—C27—C26	121.5 (2)	C42—C43—C44	120.3 (2)
C28—C27—C26	119.7 (2)	C42—C43—H43	119.8
C50—C45—P2	121.28 (18)	C13—C12—H12	119.9
C50—C45—C46	120.2 (2)	C11—C12—C13	120.2 (2)
C46—C45—P2	118.52 (18)	C11—C12—H12	119.9
C39—C44—H44	120.4	C41—C42—H42	119.8
C43—C44—C39	119.2 (2)	C43—C42—C41	120.3 (2)
C43—C44—H44	120.4	C43—C42—H42	119.8
C3—C4—H4	120.4	C48—C47—H47	119.9
C5—C4—H4	120.4	C46—C47—C48	120.1 (2)
C5—C4—C3	119.2 (2)	C46—C47—H47	119.9
C30—C29—H29	120.7	C38—C37—H37	119.9
C28—C29—H29	120.7	C36—C37—C38	120.3 (3)
C28—C29—C30	118.6 (2)	C36—C37—H37	119.9
C33—C38—H38	120.0	C12—C11—H11	119.8
C37—C38—H38	120.0	C12—C11—C10	120.3 (2)
C37—C38—C33	119.9 (2)	C10—C11—H11	119.8
C31—C30—Br2	119.21 (18)	C18—C17—H17	119.7
C31—C30—C29	121.6 (2)	C18—C17—C16	120.6 (2)
C29—C30—Br2	119.15 (18)	C16—C17—H17	119.7
C27—C28—H28	119.4	C8—C9—H9	120.2
C29—C28—C27	121.1 (2)	C10—C9—C8	119.6 (2)
C29—C28—H28	119.4	C10—C9—H9	120.2
C2—C3—H3	119.5	C11—C10—H10	119.9
C4—C3—C2	121.1 (2)	C9—C10—C11	120.2 (2)
C4—C3—H3	119.5	C9—C10—H10	119.9
C13—C8—P1	119.37 (19)	C33—C34—H34	120.2
C9—C8—P1	120.88 (19)	C35—C34—C33	119.5 (3)
C9—C8—C13	119.7 (2)	C35—C34—H34	120.2
C8—C13—H13	120.1	C15—C16—H16	120.0
C12—C13—C8	119.8 (2)	C17—C16—C15	120.1 (3)
C12—C13—H13	120.1	C17—C16—H16	120.0
C19—C14—P1	117.67 (18)	C34—C35—H35	119.9
C15—C14—P1	120.80 (18)	C36—C35—C34	120.3 (3)
C15—C14—C19	120.4 (2)	C36—C35—H35	119.9
C20—C25—H25	120.2	C37—C36—C35	120.4 (3)
C24—C25—C20	119.5 (2)	C37—C36—H36	119.8
C24—C25—H25	120.2	C35—C36—H36	119.8
C45—C50—H50	120.3	O6—Cl2A—O7	106.7 (3)
C49—C50—C45	119.4 (2)	O6—Cl2A—O5A	112.0 (4)
C49—C50—H50	120.3	O5A—Cl2A—O7	113.9 (4)
C4—C5—Br1	119.55 (17)	O8A—Cl2A—O6	107.3 (3)
C4—C5—C6	121.0 (2)	O8A—Cl2A—O7	110.9 (4)
C6—C5—Br1	119.45 (18)	O8A—Cl2A—O5A	105.9 (4)
			
Br1—C5—C6—C7	−179.96 (19)	C28—C29—C30—Br2	−179.44 (18)
P1—C20—C25—C24	179.88 (19)	C28—C29—C30—C31	0.2 (4)
P1—C20—C21—C22	−179.87 (18)	C3—C2—C1—P1	101.5 (2)
P1—C8—C13—C12	−177.7 (2)	C3—C2—C7—C6	−0.9 (4)
P1—C8—C9—C10	178.7 (2)	C3—C4—C5—Br1	179.51 (19)
P1—C14—C19—C18	−168.05 (18)	C3—C4—C5—C6	−1.0 (4)
P1—C14—C15—C16	167.6 (2)	C8—P1—C20—C25	−103.6 (2)
P2—C39—C40—C41	−171.55 (19)	C8—P1—C20—C21	76.3 (2)
P2—C39—C44—C43	170.75 (19)	C8—P1—C1—C2	63.0 (2)
P2—C45—C50—C49	−179.59 (19)	C8—P1—C14—C19	−164.17 (18)
P2—C45—C46—C47	−179.9 (2)	C8—P1—C14—C15	27.8 (2)
P2—C33—C34—C35	−179.7 (2)	C8—C13—C12—C11	−0.5 (4)
C2—C7—C6—C5	0.4 (4)	C8—C9—C10—C11	−1.5 (4)
C31—C32—C27—C28	0.7 (3)	C13—C8—C9—C10	1.1 (4)
C31—C32—C27—C26	−178.3 (2)	C13—C12—C11—C10	0.0 (4)
C20—P1—C1—C2	−177.21 (17)	C14—P1—C20—C25	16.3 (2)
C20—P1—C8—C13	−24.4 (2)	C14—P1—C20—C21	−163.86 (18)
C20—P1—C8—C9	158.0 (2)	C14—P1—C1—C2	−60.8 (2)
C20—P1—C14—C19	76.3 (2)	C14—P1—C8—C13	−141.4 (2)
C20—P1—C14—C15	−91.7 (2)	C14—P1—C8—C9	41.0 (2)
C20—C25—C24—C23	0.3 (4)	C14—C19—C18—C17	0.3 (4)
C39—P2—C45—C50	−134.2 (2)	C14—C15—C16—C17	−0.1 (4)
C39—P2—C45—C46	46.9 (2)	C25—C20—C21—C22	0.0 (3)
C39—P2—C26—C27	−48.22 (19)	C50—C45—C46—C47	1.2 (4)
C39—P2—C33—C38	−29.8 (2)	C5—C4—C3—C2	0.5 (4)
C39—P2—C33—C34	152.34 (19)	C19—C14—C15—C16	−0.1 (4)
C39—C40—C41—C42	1.3 (4)	C19—C18—C17—C16	−0.5 (4)
C39—C44—C43—C42	0.4 (4)	C26—P2—C39—C40	85.0 (2)
C1—P1—C20—C25	136.71 (19)	C26—P2—C39—C44	−85.5 (2)
C1—P1—C20—C21	−43.4 (2)	C26—P2—C45—C50	−17.7 (2)
C1—P1—C8—C13	93.9 (2)	C26—P2—C45—C46	163.36 (19)
C1—P1—C8—C9	−83.8 (2)	C26—P2—C33—C38	−146.84 (19)
C1—P1—C14—C19	−40.9 (2)	C26—P2—C33—C34	35.3 (2)
C1—P1—C14—C15	151.08 (19)	C26—C27—C28—C29	179.5 (2)
C1—C2—C3—C4	176.8 (2)	C33—P2—C39—C40	−33.8 (2)
C1—C2—C7—C6	−177.3 (2)	C33—P2—C39—C44	155.68 (18)
C32—C31—C30—Br2	−179.42 (18)	C33—P2—C45—C50	103.9 (2)
C32—C31—C30—C29	0.9 (4)	C33—P2—C45—C46	−75.0 (2)
C32—C27—C28—C29	0.5 (3)	C33—P2—C26—C27	71.02 (19)
C32—C27—C26—P2	−91.8 (2)	C33—C38—C37—C36	0.2 (4)
C40—C39—C44—C43	0.3 (4)	C33—C34—C35—C36	−0.2 (4)
C40—C41—C42—C43	−0.5 (4)	C15—C14—C19—C18	0.0 (3)
C45—P2—C39—C40	−156.49 (19)	C23—C22—C21—C20	−0.3 (4)
C45—P2—C39—C44	33.0 (2)	C22—C23—C24—C25	−0.6 (4)
C45—P2—C26—C27	−166.23 (16)	C21—C20—C25—C24	0.0 (3)
C45—P2—C33—C38	91.4 (2)	C7—C2—C1—P1	−82.1 (3)
C45—P2—C33—C34	−86.4 (2)	C7—C2—C3—C4	0.4 (4)
C45—C50—C49—C48	−0.7 (4)	C18—C17—C16—C15	0.4 (4)
C45—C46—C47—C48	−0.3 (4)	C49—C48—C47—C46	−1.0 (4)
C44—C39—C40—C41	−1.2 (4)	C24—C23—C22—C21	0.6 (4)
C44—C43—C42—C41	−0.4 (4)	C46—C45—C50—C49	−0.7 (4)
C4—C5—C6—C7	0.5 (4)	C12—C11—C10—C9	1.0 (4)
C38—C33—C34—C35	2.4 (4)	C47—C48—C49—C50	1.6 (4)
C38—C37—C36—C35	2.1 (4)	C37—C38—C33—P2	179.79 (18)
C30—C31—C32—C27	−1.4 (4)	C37—C38—C33—C34	−2.4 (3)
C30—C29—C28—C27	−0.9 (4)	C9—C8—C13—C12	−0.1 (4)
C28—C27—C26—P2	89.2 (2)	C34—C35—C36—C37	−2.0 (4)

### (4-Bromobenzyl)triphenylphosphonium perchlorate (I) Hydrogen-bond geometry (Å, º)

**Table:**

*D*—H···*A*	*D*—H	H···*A*	*D*···*A*	*D*—H···*A*
C1—H1*A*···O2	0.99	2.35	3.223 (3)	146
C1—H1*B*···O5^i^	0.99	2.48	3.443 (6)	163
C12—H12···O6^ii^	0.95	2.58	3.297 (3)	133
C17—H17···O5	0.95	2.51	3.164 (6)	126
C19—H19···O3	0.95	2.45	3.347 (4)	156
C21—H21···O5*A*^i^	0.95	2.22	3.127 (5)	159
C21—H21···O5^i^	0.95	2.30	3.238 (5)	169
C23—H23···O1^i^	0.95	2.46	3.313 (3)	149
C26—H26*A*···O2^iii^	0.99	2.36	3.327 (3)	166
C26—H26*B*···O8*A*^iv^	0.99	2.50	3.424 (5)	154
C26—H26*B*···O8^iv^	0.99	2.52	3.388 (6)	146
C31—H31···O1	0.95	2.44	3.109 (3)	127
C42—H42···O6^v^	0.95	2.47	3.414 (3)	172
C46—H46···O4^v^	0.95	2.31	3.135 (3)	145

Symmetry codes: (i) −*x*+3/2, *y*−1/2, *z*; (ii) *x*−1/2, −*y*+1/2, −*z*+1; (iii) −*x*+1, *y*+1/2, −*z*+1/2; (iv) *x*−1/2, *y*, −*z*+1/2; (v) −*x*+3/2, *y*+1/2, *z*.

### (4-Bromobenzyl)triphenylphosphonium tetrafluoroborate (II) Crystal data

**Table:**

C_25_H_21_BrP^+^·BF_4_^−^	*D*_x_ = 1.541 Mg m^−^^3^
*M**_r_* = 519.11	Cu *K*α radiation, λ = 1.54184 Å
Orthorhombic, *P**b**c**a*	Cell parameters from 23495 reflections
*a* = 15.2560 (1) Å	θ = 2.5–69.5°
*b* = 17.6466 (1) Å	µ = 3.57 mm^−^^1^
*c* = 33.2354 (3) Å	*T* = 110 K
*V* = 8947.52 (11) Å^3^	Irregular, clear colourless
*Z* = 16	0.38 × 0.28 × 0.22 mm
*F*(000) = 4192	

### (4-Bromobenzyl)triphenylphosphonium tetrafluoroborate (II) Data collection

**Table:**

XtaLAB Synergy, Single source at home/near, HyPix-Bantam diffractometer	7561 reflections with *I* > 2σ(*I*)
Detector resolution: 10.0000 pixels mm^-1^	*R*_int_ = 0.053
ω scans	θ_max_ = 69.6°, θ_min_ = 2.7°
Absorption correction: multi-scan CrysAlisPro 1.171.44.128a (Rigaku OD, 2025)	*h* = −18→14
*T*_min_ = 0.293, *T*_max_ = 1.000	*k* = −21→21
33501 measured reflections	*l* = −40→40
8341 independent reflections	

### (4-Bromobenzyl)triphenylphosphonium tetrafluoroborate (II) Refinement

**Table:**

Refinement on *F*^2^	Hydrogen site location: inferred from neighbouring sites
Least-squares matrix: full	H-atom parameters constrained
*R*[*F*^2^ > 2σ(*F*^2^)] = 0.041	*w* = 1/[σ^2^(*F*_o_^2^) + (0.0662*P*)^2^ + 8.2062*P*] where *P* = (*F*_o_^2^ + 2*F*_c_^2^)/3
*wR*(*F*^2^) = 0.111	(Δ/σ)_max_ = 0.003
*S* = 1.01	Δρ_max_ = 0.91 e Å^−^^3^
8341 reflections	Δρ_min_ = −0.68 e Å^−^^3^
597 parameters	Extinction correction: SHELXL-2018/3 (Sheldrick 2015b), Fc^*^=kFc[1+0.001xFc^2^λ^3^/sin(2θ)]^-1/4^
2 restraints	Extinction coefficient: 0.00032 (2)

### (4-Bromobenzyl)triphenylphosphonium tetrafluoroborate (II) Special details

**Table:**

Geometry. All esds (except the esd in the dihedral angle between two l.s. planes) are estimated using the full covariance matrix. The cell esds are taken into account individually in the estimation of esds in distances, angles and torsion angles; correlations between esds in cell parameters are only used when they are defined by crystal symmetry. An approximate (isotropic) treatment of cell esds is used for estimating esds involving l.s. planes.

### (4-Bromobenzyl)triphenylphosphonium tetrafluoroborate (II) Fractional atomic coordinates and isotropic or equivalent isotropic displacement parameters (Å^2^)

**Table:**

	*x*	*y*	*z*	*U*_iso_*/*U*_eq_	Occ. (<1)
Br2	0.65580 (2)	1.00767 (2)	0.51363 (2)	0.02436 (10)	
Br1	0.29893 (2)	0.59745 (2)	0.88058 (2)	0.03477 (11)	
P2	0.29173 (4)	0.75742 (3)	0.54953 (2)	0.01267 (14)	
P1	0.60960 (4)	0.75513 (3)	0.74463 (2)	0.01473 (14)	
F4	0.65224 (12)	0.38978 (10)	0.77420 (5)	0.0367 (4)	
F8	0.58688 (12)	0.53755 (9)	0.59533 (5)	0.0367 (4)	
F2	0.58832 (12)	0.32665 (9)	0.82547 (5)	0.0370 (4)	
F7	0.57909 (14)	0.66596 (9)	0.59122 (5)	0.0429 (5)	
F3	0.56640 (12)	0.45269 (10)	0.81837 (5)	0.0394 (4)	
F1	0.69785 (12)	0.40771 (10)	0.83864 (6)	0.0402 (4)	
C27	0.45084 (15)	0.83435 (13)	0.56342 (7)	0.0153 (5)	
C39	0.22366 (15)	0.69233 (12)	0.57707 (6)	0.0138 (4)	
C2	0.46132 (15)	0.67732 (14)	0.76940 (7)	0.0169 (5)	
C13	0.75159 (16)	0.71599 (14)	0.79248 (7)	0.0177 (5)	
H13	0.750269	0.766855	0.801826	0.021*	
C14	0.57982 (16)	0.82355 (13)	0.78181 (7)	0.0165 (5)	
C26	0.38642 (15)	0.77801 (13)	0.58077 (7)	0.0158 (5)	
H26A	0.365452	0.797622	0.606968	0.019*	
H26B	0.417881	0.729963	0.586057	0.019*	
C5	0.36616 (16)	0.62999 (14)	0.83563 (7)	0.0193 (5)	
C15	0.58006 (16)	0.80370 (13)	0.82255 (7)	0.0172 (5)	
H15	0.603704	0.756435	0.830886	0.021*	
C44	0.13316 (16)	0.70367 (13)	0.57941 (7)	0.0182 (5)	
H44	0.106947	0.745735	0.566235	0.022*	
C4	0.42679 (16)	0.58127 (14)	0.81884 (7)	0.0190 (5)	
H4	0.435612	0.532087	0.829729	0.023*	
C6	0.35240 (16)	0.70230 (14)	0.82050 (8)	0.0206 (5)	
H6	0.311337	0.735438	0.832841	0.025*	
C7	0.39969 (15)	0.72527 (13)	0.78705 (7)	0.0183 (5)	
H7	0.390007	0.774221	0.776019	0.022*	
C45	0.32581 (16)	0.71587 (13)	0.50285 (7)	0.0158 (5)	
C33	0.22564 (16)	0.83940 (12)	0.54019 (7)	0.0151 (5)	
C30	0.57288 (16)	0.93694 (13)	0.53390 (7)	0.0168 (5)	
C3	0.47455 (16)	0.60553 (13)	0.78578 (7)	0.0175 (5)	
H3	0.516914	0.572748	0.774119	0.021*	
C40	0.26128 (16)	0.62962 (13)	0.59632 (7)	0.0182 (5)	
H40	0.322724	0.621354	0.594810	0.022*	
C20	0.64880 (16)	0.80414 (13)	0.70087 (7)	0.0172 (5)	
C31	0.51377 (16)	0.96021 (13)	0.56305 (8)	0.0207 (5)	
H31	0.515114	1.010600	0.573093	0.025*	
C46	0.32720 (17)	0.63698 (14)	0.49883 (7)	0.0189 (5)	
H46	0.308599	0.605632	0.520439	0.023*	
C32	0.45259 (16)	0.90859 (13)	0.57732 (7)	0.0190 (5)	
H32	0.411132	0.924309	0.596947	0.023*	
C21	0.62054 (16)	0.78381 (14)	0.66249 (7)	0.0204 (5)	
H21	0.580379	0.743211	0.658957	0.025*	
C1	0.51030 (16)	0.70343 (14)	0.73228 (7)	0.0193 (5)	
H1A	0.471464	0.736360	0.716033	0.023*	
H1B	0.525717	0.658740	0.715719	0.023*	
C29	0.57310 (16)	0.86357 (14)	0.51987 (7)	0.0201 (5)	
H29	0.614614	0.848175	0.500190	0.024*	
C38	0.21203 (16)	0.88991 (13)	0.57202 (8)	0.0183 (5)	
H38	0.245968	0.886005	0.595894	0.022*	
C8	0.69064 (16)	0.69141 (13)	0.76412 (7)	0.0172 (5)	
C34	0.17597 (18)	0.84530 (14)	0.50519 (8)	0.0220 (5)	
H34	0.185325	0.810930	0.483617	0.026*	
C28	0.51206 (16)	0.81213 (13)	0.53472 (7)	0.0193 (5)	
H28	0.512176	0.761379	0.525182	0.023*	
C25	0.70942 (17)	0.86266 (14)	0.70624 (7)	0.0205 (5)	
H25	0.729617	0.875304	0.732428	0.025*	
C19	0.54346 (16)	0.89200 (14)	0.76936 (8)	0.0202 (5)	
H19	0.542770	0.905219	0.741656	0.024*	
C42	0.11936 (17)	0.59132 (14)	0.62023 (7)	0.0200 (5)	
H42	0.083683	0.557227	0.635103	0.024*	
C16	0.54555 (17)	0.85347 (15)	0.85068 (7)	0.0230 (5)	
H16	0.546560	0.840690	0.878446	0.028*	
C41	0.20842 (17)	0.57946 (14)	0.61767 (7)	0.0194 (5)	
H41	0.233927	0.536767	0.630563	0.023*	
C50	0.35342 (17)	0.76136 (14)	0.47107 (8)	0.0223 (5)	
H50	0.353298	0.814949	0.473814	0.027*	
C12	0.81426 (17)	0.66596 (16)	0.80703 (8)	0.0250 (6)	
H12	0.855476	0.682466	0.826589	0.030*	
C47	0.35600 (18)	0.60465 (14)	0.46299 (8)	0.0240 (6)	
H47	0.357413	0.551090	0.460199	0.029*	
C37	0.14842 (17)	0.94585 (14)	0.56839 (8)	0.0226 (5)	
H37	0.138942	0.980631	0.589767	0.027*	
C48	0.38246 (17)	0.65013 (15)	0.43155 (7)	0.0237 (5)	
H48	0.401711	0.627701	0.407110	0.028*	
C24	0.73969 (18)	0.90198 (14)	0.67302 (8)	0.0248 (6)	
H24	0.780902	0.941849	0.676333	0.030*	
C43	0.08181 (17)	0.65330 (14)	0.60101 (8)	0.0230 (5)	
H43	0.020321	0.661187	0.602706	0.028*	
C17	0.50960 (18)	0.92181 (15)	0.83843 (8)	0.0264 (6)	
H17	0.485931	0.955675	0.857808	0.032*	
C49	0.38118 (18)	0.72830 (15)	0.43531 (8)	0.0256 (6)	
H49	0.399262	0.759299	0.413466	0.031*	
C23	0.70971 (17)	0.88310 (15)	0.63468 (8)	0.0242 (5)	
H23	0.729181	0.911257	0.612015	0.029*	
C18	0.50808 (18)	0.94088 (14)	0.79799 (8)	0.0259 (6)	
H18	0.482775	0.987553	0.789745	0.031*	
C22	0.65191 (17)	0.82373 (16)	0.62945 (8)	0.0245 (6)	
H22	0.633472	0.810093	0.603127	0.029*	
C9	0.69502 (18)	0.61723 (15)	0.74939 (9)	0.0267 (6)	
H9	0.655061	0.600628	0.729316	0.032*	
C36	0.09884 (18)	0.95086 (14)	0.53358 (9)	0.0279 (6)	
H36	0.054738	0.988649	0.531424	0.033*	
C35	0.11285 (19)	0.90141 (15)	0.50191 (9)	0.0282 (6)	
H35	0.079253	0.905929	0.477958	0.034*	
C11	0.81679 (19)	0.59213 (16)	0.79311 (9)	0.0313 (7)	
H11	0.859149	0.557815	0.803458	0.038*	
F5A	0.5247 (6)	0.6105 (3)	0.64445 (19)	0.065 (2)	0.597 (11)
C10	0.75806 (19)	0.56818 (15)	0.76428 (9)	0.0322 (7)	
H10	0.760830	0.517626	0.754536	0.039*	
F6A	0.4648 (2)	0.5964 (2)	0.5824 (2)	0.058 (2)	0.597 (11)
B1	0.6260 (2)	0.39492 (15)	0.81402 (8)	0.0192 (6)	
B2	0.5440 (2)	0.60088 (18)	0.60777 (14)	0.0375 (9)	
F6B	0.4532 (3)	0.5915 (3)	0.6153 (3)	0.057 (3)	0.403 (11)
F5B	0.5751 (5)	0.5993 (3)	0.65225 (15)	0.0311 (15)	0.403 (11)

### (4-Bromobenzyl)triphenylphosphonium tetrafluoroborate (II) Atomic displacement parameters (Å^2^)

**Table:**

	*U*^11^	*U*^22^	*U*^33^	*U*^12^	*U*^13^	*U*^23^
Br2	0.02348 (17)	0.01706 (15)	0.03254 (17)	−0.00489 (10)	0.00785 (11)	0.00349 (10)
Br1	0.0444 (2)	0.02063 (17)	0.03922 (19)	−0.00359 (12)	0.02275 (14)	0.00192 (12)
P2	0.0166 (3)	0.0067 (3)	0.0148 (3)	−0.0014 (2)	0.0003 (2)	0.0003 (2)
P1	0.0189 (3)	0.0106 (3)	0.0147 (3)	−0.0008 (2)	−0.0001 (2)	−0.0009 (2)
F4	0.0453 (10)	0.0424 (10)	0.0224 (8)	0.0017 (8)	0.0091 (7)	−0.0037 (7)
F8	0.0604 (12)	0.0169 (8)	0.0330 (8)	0.0083 (8)	0.0129 (8)	0.0013 (7)
F2	0.0493 (10)	0.0217 (8)	0.0400 (9)	−0.0096 (8)	0.0049 (8)	0.0001 (7)
F7	0.0740 (13)	0.0164 (8)	0.0384 (9)	−0.0059 (8)	−0.0156 (9)	0.0010 (7)
F3	0.0479 (10)	0.0287 (9)	0.0415 (9)	0.0208 (8)	−0.0020 (8)	−0.0071 (7)
F1	0.0379 (10)	0.0411 (10)	0.0415 (10)	−0.0023 (8)	−0.0183 (8)	−0.0048 (8)
C27	0.0166 (11)	0.0109 (10)	0.0184 (11)	−0.0009 (9)	−0.0033 (9)	0.0016 (9)
C39	0.0200 (11)	0.0090 (10)	0.0123 (10)	−0.0039 (9)	0.0016 (9)	−0.0013 (8)
C2	0.0174 (11)	0.0164 (11)	0.0169 (11)	−0.0036 (9)	−0.0034 (9)	−0.0004 (9)
C13	0.0185 (12)	0.0165 (11)	0.0182 (11)	0.0006 (10)	0.0044 (9)	0.0038 (9)
C14	0.0167 (11)	0.0132 (11)	0.0196 (11)	−0.0024 (9)	0.0005 (9)	−0.0011 (9)
C26	0.0171 (11)	0.0114 (10)	0.0189 (11)	−0.0003 (9)	−0.0028 (9)	0.0007 (9)
C5	0.0192 (12)	0.0164 (11)	0.0222 (12)	−0.0028 (10)	0.0026 (10)	−0.0001 (10)
C15	0.0184 (11)	0.0151 (11)	0.0179 (11)	0.0009 (9)	0.0000 (9)	0.0005 (9)
C44	0.0208 (12)	0.0125 (11)	0.0213 (11)	−0.0001 (10)	0.0019 (10)	0.0005 (9)
C4	0.0200 (12)	0.0143 (11)	0.0225 (11)	−0.0006 (10)	−0.0045 (10)	0.0011 (10)
C6	0.0173 (12)	0.0158 (12)	0.0287 (13)	0.0015 (10)	0.0002 (10)	−0.0013 (10)
C7	0.0170 (11)	0.0140 (11)	0.0239 (12)	−0.0009 (9)	−0.0048 (10)	0.0026 (9)
C45	0.0159 (11)	0.0137 (11)	0.0177 (11)	−0.0026 (9)	−0.0006 (9)	−0.0017 (9)
C33	0.0187 (11)	0.0073 (10)	0.0192 (11)	−0.0026 (9)	0.0001 (9)	0.0024 (9)
C30	0.0201 (12)	0.0110 (10)	0.0193 (11)	−0.0039 (9)	0.0003 (10)	0.0046 (9)
C3	0.0175 (11)	0.0148 (11)	0.0203 (11)	−0.0005 (9)	−0.0003 (10)	−0.0025 (9)
C40	0.0196 (12)	0.0149 (11)	0.0200 (11)	−0.0012 (10)	−0.0005 (10)	−0.0002 (9)
C20	0.0203 (12)	0.0134 (11)	0.0178 (11)	0.0006 (9)	0.0008 (9)	−0.0003 (9)
C31	0.0216 (12)	0.0111 (11)	0.0293 (13)	−0.0017 (10)	0.0050 (11)	−0.0049 (10)
C46	0.0234 (13)	0.0133 (11)	0.0201 (11)	0.0001 (10)	0.0025 (10)	0.0007 (10)
C32	0.0210 (12)	0.0138 (11)	0.0224 (12)	0.0005 (10)	0.0041 (10)	−0.0014 (9)
C21	0.0206 (12)	0.0209 (12)	0.0198 (11)	−0.0029 (10)	0.0005 (10)	−0.0030 (10)
C1	0.0224 (12)	0.0175 (11)	0.0180 (11)	−0.0048 (10)	−0.0026 (10)	−0.0003 (9)
C29	0.0177 (12)	0.0196 (12)	0.0230 (12)	−0.0006 (10)	0.0041 (10)	−0.0054 (10)
C38	0.0205 (12)	0.0109 (11)	0.0234 (12)	−0.0024 (9)	0.0010 (10)	−0.0001 (9)
C8	0.0216 (12)	0.0104 (11)	0.0197 (11)	0.0017 (9)	0.0047 (10)	0.0022 (9)
C34	0.0288 (14)	0.0157 (12)	0.0215 (12)	−0.0023 (11)	−0.0031 (11)	0.0022 (10)
C28	0.0218 (12)	0.0113 (10)	0.0250 (12)	−0.0010 (10)	0.0009 (10)	−0.0038 (9)
C25	0.0228 (12)	0.0179 (12)	0.0207 (12)	−0.0014 (10)	0.0000 (10)	−0.0016 (10)
C19	0.0238 (12)	0.0162 (12)	0.0207 (12)	0.0000 (10)	0.0015 (10)	0.0014 (10)
C42	0.0265 (13)	0.0141 (11)	0.0193 (12)	−0.0074 (10)	0.0064 (10)	−0.0009 (9)
C16	0.0264 (13)	0.0242 (13)	0.0183 (11)	−0.0006 (11)	0.0045 (10)	−0.0025 (10)
C41	0.0287 (13)	0.0109 (11)	0.0187 (11)	−0.0023 (10)	0.0001 (10)	0.0021 (9)
C50	0.0301 (14)	0.0152 (12)	0.0216 (12)	−0.0062 (10)	0.0040 (11)	0.0011 (10)
C12	0.0206 (12)	0.0302 (14)	0.0242 (12)	0.0037 (11)	0.0052 (10)	0.0113 (11)
C47	0.0284 (14)	0.0165 (12)	0.0273 (13)	−0.0005 (10)	0.0033 (11)	−0.0068 (10)
C37	0.0242 (13)	0.0102 (11)	0.0334 (14)	0.0003 (10)	0.0019 (11)	−0.0005 (10)
C48	0.0236 (13)	0.0270 (14)	0.0205 (12)	−0.0036 (11)	0.0057 (10)	−0.0078 (11)
C24	0.0274 (14)	0.0164 (12)	0.0304 (13)	−0.0050 (10)	0.0058 (12)	−0.0009 (10)
C43	0.0200 (12)	0.0182 (12)	0.0309 (13)	−0.0020 (10)	0.0065 (11)	0.0003 (11)
C17	0.0296 (14)	0.0189 (12)	0.0308 (13)	0.0019 (11)	0.0089 (12)	−0.0085 (11)
C49	0.0308 (14)	0.0252 (13)	0.0208 (12)	−0.0077 (12)	0.0080 (11)	0.0005 (10)
C23	0.0275 (13)	0.0207 (13)	0.0243 (12)	0.0021 (11)	0.0070 (11)	0.0039 (11)
C18	0.0297 (14)	0.0144 (12)	0.0335 (14)	0.0042 (11)	0.0064 (12)	0.0032 (11)
C22	0.0269 (14)	0.0294 (14)	0.0171 (11)	0.0019 (11)	0.0010 (10)	0.0026 (11)
C9	0.0289 (14)	0.0152 (12)	0.0361 (15)	−0.0026 (11)	0.0069 (12)	−0.0037 (11)
C36	0.0248 (14)	0.0123 (11)	0.0466 (16)	0.0033 (10)	−0.0029 (12)	0.0070 (12)
C35	0.0292 (15)	0.0225 (13)	0.0329 (14)	0.0016 (11)	−0.0121 (12)	0.0064 (11)
C11	0.0272 (14)	0.0235 (14)	0.0431 (17)	0.0111 (12)	0.0120 (13)	0.0157 (12)
F5A	0.104 (6)	0.030 (2)	0.060 (3)	−0.017 (3)	0.056 (4)	−0.018 (2)
C10	0.0341 (15)	0.0129 (12)	0.0496 (17)	0.0053 (11)	0.0176 (14)	0.0018 (12)
F6A	0.0259 (17)	0.039 (2)	0.108 (5)	−0.0023 (14)	−0.005 (2)	−0.025 (2)
B1	0.0241 (14)	0.0143 (12)	0.0191 (13)	0.0018 (11)	−0.0022 (11)	−0.0041 (10)
B2	0.0258 (16)	0.0146 (14)	0.072 (3)	−0.0026 (12)	0.0037 (17)	−0.0138 (16)
F6B	0.023 (3)	0.044 (3)	0.105 (8)	0.001 (2)	−0.007 (3)	−0.005 (3)
F5B	0.047 (4)	0.021 (2)	0.024 (2)	−0.006 (2)	0.004 (2)	−0.0034 (17)

### (4-Bromobenzyl)triphenylphosphonium tetrafluoroborate (II) Geometric parameters (Å, º)

**Table:**

Br2—C30	1.901 (2)	C46—C47	1.392 (4)
Br1—C5	1.901 (2)	C32—H32	0.9500
P2—C39	1.799 (2)	C21—H21	0.9500
P2—C26	1.816 (2)	C21—C22	1.390 (4)
P2—C45	1.793 (2)	C1—H1A	0.9900
P2—C33	1.791 (2)	C1—H1B	0.9900
P1—C14	1.787 (2)	C29—H29	0.9500
P1—C20	1.795 (2)	C29—C28	1.391 (3)
P1—C1	1.815 (2)	C38—H38	0.9500
P1—C8	1.792 (2)	C38—C37	1.390 (3)
F4—B1	1.385 (3)	C8—C9	1.399 (4)
F8—B2	1.360 (4)	C34—H34	0.9500
F2—B1	1.388 (3)	C34—C35	1.386 (4)
F7—B2	1.381 (4)	C28—H28	0.9500
F3—B1	1.374 (3)	C25—H25	0.9500
F1—B1	1.386 (3)	C25—C24	1.383 (4)
C27—C26	1.512 (3)	C19—H19	0.9500
C27—C32	1.390 (3)	C19—C18	1.393 (4)
C27—C28	1.391 (3)	C42—H42	0.9500
C39—C44	1.397 (3)	C42—C41	1.377 (4)
C39—C40	1.401 (3)	C42—C43	1.390 (4)
C2—C7	1.394 (3)	C16—H16	0.9500
C2—C3	1.393 (3)	C16—C17	1.386 (4)
C2—C1	1.514 (3)	C41—H41	0.9500
C13—H13	0.9500	C50—H50	0.9500
C13—C8	1.393 (4)	C50—C49	1.390 (4)
C13—C12	1.388 (4)	C12—H12	0.9500
C14—C15	1.398 (3)	C12—C11	1.383 (4)
C14—C19	1.392 (3)	C47—H47	0.9500
C26—H26A	0.9900	C47—C48	1.378 (4)
C26—H26B	0.9900	C37—H37	0.9500
C5—C4	1.381 (4)	C37—C36	1.385 (4)
C5—C6	1.387 (3)	C48—H48	0.9500
C15—H15	0.9500	C48—C49	1.385 (4)
C15—C16	1.387 (3)	C24—H24	0.9500
C44—H44	0.9500	C24—C23	1.394 (4)
C44—C43	1.385 (3)	C43—H43	0.9500
C4—H4	0.9500	C17—H17	0.9500
C4—C3	1.386 (3)	C17—C18	1.386 (4)
C6—H6	0.9500	C49—H49	0.9500
C6—C7	1.386 (4)	C23—H23	0.9500
C7—H7	0.9500	C23—C22	1.380 (4)
C45—C46	1.399 (3)	C18—H18	0.9500
C45—C50	1.392 (3)	C22—H22	0.9500
C33—C38	1.399 (3)	C9—H9	0.9500
C33—C34	1.392 (3)	C9—C10	1.385 (4)
C30—C31	1.386 (3)	C36—H36	0.9500
C30—C29	1.376 (3)	C36—C35	1.384 (4)
C3—H3	0.9500	C35—H35	0.9500
C40—H40	0.9500	C11—H11	0.9500
C40—C41	1.392 (3)	C11—C10	1.378 (5)
C20—C21	1.393 (3)	F5A—B2	1.265 (6)
C20—C25	1.398 (3)	C10—H10	0.9500
C31—H31	0.9500	F6A—B2	1.475 (6)
C31—C32	1.388 (3)	B2—F6B	1.417 (7)
C46—H46	0.9500	B2—F5B	1.553 (7)
			
C39—P2—C26	107.23 (11)	C13—C8—P1	120.63 (18)
C45—P2—C39	110.28 (11)	C13—C8—C9	119.8 (2)
C45—P2—C26	110.24 (11)	C9—C8—P1	119.6 (2)
C33—P2—C39	106.20 (11)	C33—C34—H34	120.1
C33—P2—C26	112.67 (11)	C35—C34—C33	119.8 (2)
C33—P2—C45	110.10 (11)	C35—C34—H34	120.1
C14—P1—C20	108.65 (11)	C27—C28—H28	119.7
C14—P1—C1	106.49 (11)	C29—C28—C27	120.6 (2)
C14—P1—C8	110.45 (11)	C29—C28—H28	119.7
C20—P1—C1	109.69 (11)	C20—C25—H25	120.3
C8—P1—C20	111.43 (11)	C24—C25—C20	119.3 (2)
C8—P1—C1	110.00 (12)	C24—C25—H25	120.3
C32—C27—C26	120.4 (2)	C14—C19—H19	120.4
C32—C27—C28	118.7 (2)	C14—C19—C18	119.2 (2)
C28—C27—C26	120.8 (2)	C18—C19—H19	120.4
C44—C39—P2	120.49 (18)	C41—C42—H42	120.1
C44—C39—C40	119.5 (2)	C41—C42—C43	119.9 (2)
C40—C39—P2	120.01 (18)	C43—C42—H42	120.1
C7—C2—C1	119.4 (2)	C15—C16—H16	119.9
C3—C2—C7	119.0 (2)	C17—C16—C15	120.2 (2)
C3—C2—C1	121.6 (2)	C17—C16—H16	119.9
C8—C13—H13	120.1	C40—C41—H41	119.8
C12—C13—H13	120.1	C42—C41—C40	120.4 (2)
C12—C13—C8	119.8 (2)	C42—C41—H41	119.8
C15—C14—P1	119.98 (18)	C45—C50—H50	120.0
C19—C14—P1	118.82 (18)	C49—C50—C45	119.9 (2)
C19—C14—C15	120.4 (2)	C49—C50—H50	120.0
P2—C26—H26A	108.4	C13—C12—H12	119.9
P2—C26—H26B	108.4	C11—C12—C13	120.1 (3)
C27—C26—P2	115.50 (16)	C11—C12—H12	119.9
C27—C26—H26A	108.4	C46—C47—H47	119.9
C27—C26—H26B	108.4	C48—C47—C46	120.2 (2)
H26A—C26—H26B	107.5	C48—C47—H47	119.9
C4—C5—Br1	119.41 (18)	C38—C37—H37	120.0
C4—C5—C6	121.8 (2)	C36—C37—C38	120.0 (2)
C6—C5—Br1	118.75 (19)	C36—C37—H37	120.0
C14—C15—H15	120.2	C47—C48—H48	119.8
C16—C15—C14	119.5 (2)	C47—C48—C49	120.5 (2)
C16—C15—H15	120.2	C49—C48—H48	119.8
C39—C44—H44	120.1	C25—C24—H24	120.0
C43—C44—C39	119.7 (2)	C25—C24—C23	120.0 (2)
C43—C44—H44	120.1	C23—C24—H24	120.0
C5—C4—H4	120.6	C44—C43—C42	120.7 (2)
C5—C4—C3	118.7 (2)	C44—C43—H43	119.7
C3—C4—H4	120.6	C42—C43—H43	119.7
C5—C6—H6	120.6	C16—C17—H17	119.9
C7—C6—C5	118.7 (2)	C18—C17—C16	120.2 (2)
C7—C6—H6	120.6	C18—C17—H17	119.9
C2—C7—H7	119.6	C50—C49—H49	120.0
C6—C7—C2	120.7 (2)	C48—C49—C50	120.0 (2)
C6—C7—H7	119.6	C48—C49—H49	120.0
C46—C45—P2	119.61 (18)	C24—C23—H23	119.8
C50—C45—P2	120.57 (19)	C22—C23—C24	120.4 (2)
C50—C45—C46	119.8 (2)	C22—C23—H23	119.8
C38—C33—P2	117.86 (18)	C19—C18—H18	119.8
C34—C33—P2	120.79 (18)	C17—C18—C19	120.4 (2)
C34—C33—C38	120.2 (2)	C17—C18—H18	119.8
C31—C30—Br2	119.10 (18)	C21—C22—H22	119.8
C29—C30—Br2	119.74 (18)	C23—C22—C21	120.4 (2)
C29—C30—C31	121.2 (2)	C23—C22—H22	119.8
C2—C3—H3	119.5	C8—C9—H9	120.2
C4—C3—C2	120.9 (2)	C10—C9—C8	119.5 (3)
C4—C3—H3	119.5	C10—C9—H9	120.2
C39—C40—H40	120.1	C37—C36—H36	119.6
C41—C40—C39	119.8 (2)	C35—C36—C37	120.7 (2)
C41—C40—H40	120.1	C35—C36—H36	119.6
C21—C20—P1	120.98 (19)	C34—C35—H35	120.1
C21—C20—C25	120.8 (2)	C36—C35—C34	119.9 (3)
C25—C20—P1	118.23 (18)	C36—C35—H35	120.1
C30—C31—H31	120.6	C12—C11—H11	119.9
C30—C31—C32	118.8 (2)	C10—C11—C12	120.2 (3)
C32—C31—H31	120.6	C10—C11—H11	119.9
C45—C46—H46	120.2	C9—C10—H10	119.7
C47—C46—C45	119.6 (2)	C11—C10—C9	120.5 (3)
C47—C46—H46	120.2	C11—C10—H10	119.7
C27—C32—H32	119.4	F4—B1—F2	108.9 (2)
C31—C32—C27	121.2 (2)	F4—B1—F1	110.3 (2)
C31—C32—H32	119.4	F3—B1—F4	109.9 (2)
C20—C21—H21	120.5	F3—B1—F2	109.9 (2)
C22—C21—C20	119.1 (2)	F3—B1—F1	109.9 (2)
C22—C21—H21	120.5	F1—B1—F2	107.9 (2)
P1—C1—H1A	109.1	F8—B2—F7	112.0 (3)
P1—C1—H1B	109.1	F8—B2—F6A	100.1 (3)
C2—C1—P1	112.36 (16)	F8—B2—F6B	115.4 (3)
C2—C1—H1A	109.1	F8—B2—F5B	97.3 (4)
C2—C1—H1B	109.1	F7—B2—F6A	97.7 (3)
H1A—C1—H1B	107.9	F7—B2—F6B	123.2 (4)
C30—C29—H29	120.3	F7—B2—F5B	105.9 (3)
C30—C29—C28	119.5 (2)	F5A—B2—F8	121.0 (5)
C28—C29—H29	120.3	F5A—B2—F7	111.3 (3)
C33—C38—H38	120.3	F5A—B2—F6A	111.6 (5)
C37—C38—C33	119.4 (2)	F6B—B2—F5B	97.4 (5)
C37—C38—H38	120.3		
			
Br2—C30—C31—C32	179.09 (19)	C33—P2—C26—C27	−61.5 (2)
Br2—C30—C29—C28	−179.78 (19)	C33—P2—C45—C46	−140.1 (2)
Br1—C5—C4—C3	179.24 (18)	C33—P2—C45—C50	41.9 (2)
Br1—C5—C6—C7	−178.24 (18)	C33—C38—C37—C36	0.4 (4)
P2—C39—C44—C43	−179.49 (19)	C33—C34—C35—C36	−0.7 (4)
P2—C39—C40—C41	179.87 (18)	C30—C31—C32—C27	1.1 (4)
P2—C45—C46—C47	−178.2 (2)	C30—C29—C28—C27	0.3 (4)
P2—C45—C50—C49	178.8 (2)	C3—C2—C7—C6	0.3 (4)
P2—C33—C38—C37	−167.83 (19)	C3—C2—C1—P1	−93.5 (2)
P2—C33—C34—C35	167.6 (2)	C40—C39—C44—C43	0.6 (3)
P1—C14—C15—C16	−171.20 (19)	C20—P1—C14—C15	−155.1 (2)
P1—C14—C19—C18	170.5 (2)	C20—P1—C14—C19	35.0 (2)
P1—C20—C21—C22	179.39 (19)	C20—P1—C1—C2	−165.56 (17)
P1—C20—C25—C24	−179.1 (2)	C20—P1—C8—C13	89.3 (2)
P1—C8—C9—C10	179.6 (2)	C20—P1—C8—C9	−87.9 (2)
C39—P2—C26—C27	−178.01 (17)	C20—C21—C22—C23	−0.5 (4)
C39—P2—C45—C46	−23.2 (2)	C20—C25—C24—C23	0.0 (4)
C39—P2—C45—C50	158.8 (2)	C31—C30—C29—C28	1.0 (4)
C39—P2—C33—C38	76.8 (2)	C46—C45—C50—C49	0.8 (4)
C39—P2—C33—C34	−91.1 (2)	C46—C47—C48—C49	0.4 (4)
C39—C44—C43—C42	−0.3 (4)	C32—C27—C26—P2	103.1 (2)
C39—C40—C41—C42	−0.4 (4)	C32—C27—C28—C29	−0.8 (4)
C13—C8—C9—C10	2.3 (4)	C21—C20—C25—C24	1.7 (4)
C13—C12—C11—C10	1.0 (4)	C1—P1—C14—C15	86.8 (2)
C14—P1—C20—C21	−134.0 (2)	C1—P1—C14—C19	−83.1 (2)
C14—P1—C20—C25	46.9 (2)	C1—P1—C20—C21	−17.9 (2)
C14—P1—C1—C2	−48.2 (2)	C1—P1—C20—C25	162.9 (2)
C14—P1—C8—C13	−31.6 (2)	C1—P1—C8—C13	−148.86 (19)
C14—P1—C8—C9	151.2 (2)	C1—P1—C8—C9	33.9 (2)
C14—C15—C16—C17	1.2 (4)	C1—C2—C7—C6	178.5 (2)
C14—C19—C18—C17	0.4 (4)	C1—C2—C3—C4	−177.5 (2)
C26—P2—C39—C44	134.36 (19)	C29—C30—C31—C32	−1.7 (4)
C26—P2—C39—C40	−45.8 (2)	C38—C33—C34—C35	0.0 (4)
C26—P2—C45—C46	95.0 (2)	C38—C37—C36—C35	−1.1 (4)
C26—P2—C45—C50	−83.0 (2)	C8—P1—C14—C15	−32.6 (2)
C26—P2—C33—C38	−40.3 (2)	C8—P1—C14—C19	157.46 (19)
C26—P2—C33—C34	151.8 (2)	C8—P1—C20—C21	104.1 (2)
C26—C27—C32—C31	177.1 (2)	C8—P1—C20—C25	−75.0 (2)
C26—C27—C28—C29	−177.8 (2)	C8—P1—C1—C2	71.5 (2)
C5—C4—C3—C2	−0.7 (4)	C8—C13—C12—C11	0.7 (4)
C5—C6—C7—C2	−1.4 (4)	C8—C9—C10—C11	−0.6 (4)
C15—C14—C19—C18	0.6 (4)	C34—C33—C38—C37	0.1 (4)
C15—C16—C17—C18	−0.1 (4)	C28—C27—C26—P2	−80.0 (3)
C44—C39—C40—C41	−0.2 (3)	C28—C27—C32—C31	0.1 (4)
C4—C5—C6—C7	1.4 (4)	C25—C20—C21—C22	−1.5 (4)
C6—C5—C4—C3	−0.4 (4)	C25—C24—C23—C22	−2.0 (4)
C7—C2—C3—C4	0.7 (4)	C19—C14—C15—C16	−1.5 (4)
C7—C2—C1—P1	88.3 (2)	C16—C17—C18—C19	−0.7 (4)
C45—P2—C39—C44	−105.6 (2)	C41—C42—C43—C44	−0.3 (4)
C45—P2—C39—C40	74.3 (2)	C50—C45—C46—C47	−0.2 (4)
C45—P2—C26—C27	61.9 (2)	C12—C13—C8—P1	−179.56 (18)
C45—P2—C33—C38	−163.83 (18)	C12—C13—C8—C9	−2.4 (4)
C45—P2—C33—C34	28.3 (2)	C12—C11—C10—C9	−1.0 (4)
C45—C46—C47—C48	−0.4 (4)	C47—C48—C49—C50	0.3 (4)
C45—C50—C49—C48	−0.9 (4)	C37—C36—C35—C34	1.2 (4)
C33—P2—C39—C44	13.7 (2)	C24—C23—C22—C21	2.3 (4)
C33—P2—C39—C40	−166.44 (18)	C43—C42—C41—C40	0.7 (4)

### (4-Bromobenzyl)triphenylphosphonium tetrafluoroborate (II) Hydrogen-bond geometry (Å, º)

**Table:**

*D*—H···*A*	*D*—H	H···*A*	*D*···*A*	*D*—H···*A*
C1—H1*A*···F2^i^	0.99	2.30	3.267 (3)	167
C1—H1*B*···F5*A*	0.99	2.52	3.355 (7)	142
C1—H1*B*···F5*B*	0.99	2.47	3.381 (6)	152
C4—H4···F3	0.95	2.47	3.112 (3)	125
C17—H17···F8^i^	0.95	2.40	3.344 (3)	175
C21—H21···F5*A*	0.95	2.54	3.443 (7)	159
C25—H25···F4^ii^	0.95	2.29	3.128 (3)	147
C26—H26*A*···F2^i^	0.99	2.41	3.255 (3)	143
C26—H26*B*···F6*A*	0.99	2.47	3.421 (4)	162
C38—H38···F1^i^	0.95	2.37	3.287 (3)	162
C40—H40···F6*A*	0.95	2.25	3.193 (4)	172
C40—H40···F6*B*	0.95	2.17	3.070 (6)	158
C42—H42···F3^iii^	0.95	2.42	3.287 (3)	151

Symmetry codes: (i) −*x*+1, *y*+1/2, −*z*+3/2; (ii) −*x*+3/2, *y*+1/2, *z*; (iii) *x*−1/2, *y*, −*z*+3/2.

### (4-Bromobenzyl)triphenylphosphonium triiodide (III) Crystal data

**Table:**

C_25_H_21_BrP^+^·I_3_^−^	*F*(000) = 1520
*M**_r_* = 813.00	*D*_x_ = 2.061 Mg m^−^^3^
Monoclinic, *P*2_1_/*n*	Cu *K*α radiation, λ = 1.54184 Å
*a* = 9.8825 (1) Å	Cell parameters from 10954 reflections
*b* = 14.8840 (2) Å	θ = 2.5–69.6°
*c* = 17.8209 (2) Å	µ = 30.54 mm^−^^1^
β = 91.504 (1)°	*T* = 111 K
*V* = 2620.39 (5) Å^3^	Rect. Prism, clear dark red
*Z* = 4	0.12 × 0.10 × 0.04 mm

### (4-Bromobenzyl)triphenylphosphonium triiodide (III) Data collection

**Table:**

XtaLAB Synergy, Single source at home/near, HyPix-Bantam diffractometer	4462 reflections with *I* > 2σ(*I*)
Detector resolution: 10.0000 pixels mm^-1^	*R*_int_ = 0.095
ω scans	θ_max_ = 69.6°, θ_min_ = 3.9°
Absorption correction: multi-scan CrysAlisPro 1.171.44.128a (Rigaku OD, 2025)	*h* = −12→11
*T*_min_ = 0.233, *T*_max_ = 1.000	*k* = −18→13
13993 measured reflections	*l* = −21→21
4888 independent reflections	

### (4-Bromobenzyl)triphenylphosphonium triiodide (III) Refinement

**Table:**

Refinement on *F*^2^	Primary atom site location: dual
Least-squares matrix: full	Hydrogen site location: inferred from neighbouring sites
*R*[*F*^2^ > 2σ(*F*^2^)] = 0.044	H-atom parameters constrained
*wR*(*F*^2^) = 0.123	*w* = 1/[σ^2^(*F*_o_^2^) + (0.0863*P*)^2^] where *P* = (*F*_o_^2^ + 2*F*_c_^2^)/3
*S* = 1.03	(Δ/σ)_max_ = 0.001
4888 reflections	Δρ_max_ = 1.27 e Å^−^^3^
271 parameters	Δρ_min_ = −1.71 e Å^−^^3^
0 restraints	

### (4-Bromobenzyl)triphenylphosphonium triiodide (III) Special details

**Table:**

Geometry. All esds (except the esd in the dihedral angle between two l.s. planes) are estimated using the full covariance matrix. The cell esds are taken into account individually in the estimation of esds in distances, angles and torsion angles; correlations between esds in cell parameters are only used when they are defined by crystal symmetry. An approximate (isotropic) treatment of cell esds is used for estimating esds involving l.s. planes.

### (4-Bromobenzyl)triphenylphosphonium triiodide (III) Fractional atomic coordinates and isotropic or equivalent isotropic displacement parameters (Å^2^)

**Table:**

	*x*	*y*	*z*	*U*_iso_*/*U*_eq_	
I2	0.73689 (3)	0.25197 (2)	0.43478 (2)	0.02207 (13)	
I1	0.78470 (3)	0.44287 (2)	0.38645 (2)	0.02570 (13)	
I3	0.66032 (4)	0.07646 (3)	0.48339 (2)	0.03540 (14)	
Br1	0.15259 (6)	0.13923 (4)	0.43969 (3)	0.02601 (16)	
P1	0.34696 (12)	0.58128 (9)	0.32740 (6)	0.0174 (3)	
C8	0.4020 (5)	0.5099 (3)	0.2533 (2)	0.0210 (10)	
C6	0.3519 (5)	0.2728 (4)	0.4130 (3)	0.0216 (10)	
H6	0.411047	0.223417	0.405794	0.026*	
C5	0.2170 (5)	0.2577 (4)	0.4294 (3)	0.0221 (11)	
C18	0.5025 (6)	0.8179 (4)	0.2589 (3)	0.0286 (12)	
H18	0.496897	0.853580	0.214693	0.034*	
C19	0.4292 (6)	0.7388 (4)	0.2637 (3)	0.0286 (12)	
H19	0.369631	0.721602	0.223510	0.034*	
C14	0.4422 (5)	0.6844 (4)	0.3267 (2)	0.0193 (10)	
C2	0.3157 (5)	0.4326 (4)	0.4196 (2)	0.0199 (10)	
C4	0.1314 (5)	0.3304 (4)	0.4397 (2)	0.0220 (10)	
H4	0.038647	0.320623	0.449830	0.026*	
C15	0.5236 (6)	0.7111 (4)	0.3873 (3)	0.0286 (12)	
H15	0.530842	0.674857	0.431060	0.034*	
C13	0.5234 (6)	0.5274 (4)	0.2181 (3)	0.0304 (12)	
H13	0.578469	0.576668	0.233566	0.036*	
C9	0.3227 (7)	0.4366 (4)	0.2320 (3)	0.0319 (13)	
H9	0.240943	0.424445	0.256946	0.038*	
C17	0.5836 (6)	0.8442 (4)	0.3192 (3)	0.0299 (12)	
H17	0.632512	0.899072	0.316698	0.036*	
C1	0.3747 (5)	0.5257 (3)	0.4174 (2)	0.0187 (10)	
H1A	0.473240	0.522146	0.428425	0.022*	
H1B	0.333812	0.562504	0.457128	0.022*	
C3	0.1810 (5)	0.4169 (4)	0.4352 (3)	0.0210 (10)	
H3	0.122152	0.466368	0.442891	0.025*	
C16	0.5947 (6)	0.7919 (4)	0.3831 (3)	0.0340 (13)	
H16	0.650773	0.810933	0.424161	0.041*	
C11	0.4827 (8)	0.4002 (5)	0.1382 (3)	0.0460 (17)	
H11	0.509325	0.363228	0.097753	0.055*	
C20	0.1700 (5)	0.6064 (4)	0.3136 (3)	0.0201 (10)	
C7	0.3997 (5)	0.3593 (4)	0.4073 (3)	0.0230 (11)	
H7	0.491375	0.369175	0.394695	0.028*	
C25	0.0961 (5)	0.6397 (4)	0.3722 (3)	0.0237 (11)	
H25	0.137722	0.645180	0.420695	0.028*	
C23	−0.0980 (6)	0.6592 (4)	0.2905 (3)	0.0324 (13)	
H23	−0.189394	0.677380	0.282818	0.039*	
C24	−0.0373 (5)	0.6651 (4)	0.3613 (3)	0.0287 (11)	
H24	−0.087500	0.686601	0.402330	0.034*	
C22	−0.0249 (6)	0.6265 (4)	0.2307 (3)	0.0320 (13)	
H22	−0.066448	0.622310	0.182179	0.038*	
C12	0.5630 (7)	0.4713 (5)	0.1597 (3)	0.0398 (16)	
H12	0.645371	0.482375	0.135035	0.048*	
C10	0.3633 (8)	0.3813 (4)	0.1743 (3)	0.0407 (15)	
H10	0.309913	0.330905	0.159615	0.049*	
C21	0.1094 (5)	0.5999 (4)	0.2420 (3)	0.0282 (12)	
H21	0.159466	0.577561	0.201200	0.034*	

### (4-Bromobenzyl)triphenylphosphonium triiodide (III) Atomic displacement parameters (Å^2^)

**Table:**

	*U*^11^	*U*^22^	*U*^33^	*U*^12^	*U*^13^	*U*^23^
I2	0.0178 (2)	0.0267 (2)	0.0216 (2)	0.00089 (11)	−0.00003 (13)	−0.00543 (12)
I1	0.0229 (2)	0.0275 (2)	0.0272 (2)	−0.00270 (11)	0.00926 (13)	−0.00359 (13)
I3	0.0496 (3)	0.0265 (2)	0.0302 (2)	0.00417 (15)	0.00367 (17)	0.00381 (14)
Br1	0.0341 (3)	0.0231 (3)	0.0209 (3)	−0.0065 (2)	0.0006 (2)	0.0014 (2)
P1	0.0193 (6)	0.0218 (7)	0.0111 (5)	−0.0020 (4)	0.0017 (4)	0.0024 (5)
C8	0.033 (3)	0.018 (3)	0.011 (2)	0.004 (2)	0.0046 (18)	−0.0005 (18)
C6	0.023 (2)	0.019 (3)	0.022 (2)	0.0036 (19)	0.0036 (18)	0.002 (2)
C5	0.026 (3)	0.028 (3)	0.013 (2)	−0.005 (2)	−0.0025 (19)	0.0042 (19)
C18	0.034 (3)	0.030 (3)	0.022 (2)	−0.006 (2)	0.000 (2)	0.011 (2)
C19	0.032 (3)	0.035 (3)	0.019 (3)	−0.009 (2)	−0.003 (2)	0.008 (2)
C14	0.022 (2)	0.021 (3)	0.015 (2)	−0.0017 (18)	0.0063 (17)	0.0000 (19)
C2	0.024 (3)	0.027 (3)	0.008 (2)	−0.0006 (19)	−0.0003 (17)	−0.0010 (18)
C4	0.017 (2)	0.035 (3)	0.014 (2)	−0.003 (2)	0.0031 (17)	0.004 (2)
C15	0.030 (3)	0.038 (4)	0.017 (2)	−0.009 (2)	0.000 (2)	0.000 (2)
C13	0.032 (3)	0.035 (3)	0.024 (3)	0.007 (2)	0.003 (2)	0.014 (2)
C9	0.049 (4)	0.028 (3)	0.019 (2)	0.000 (2)	0.008 (2)	−0.005 (2)
C17	0.027 (3)	0.026 (3)	0.037 (3)	−0.007 (2)	0.003 (2)	0.002 (2)
C1	0.023 (2)	0.022 (3)	0.011 (2)	−0.0010 (18)	0.0016 (17)	0.0063 (18)
C3	0.020 (2)	0.027 (3)	0.016 (2)	0.0039 (19)	0.0038 (18)	0.0006 (19)
C16	0.039 (3)	0.036 (4)	0.027 (3)	−0.015 (3)	−0.007 (2)	0.001 (2)
C11	0.073 (5)	0.042 (4)	0.024 (3)	0.013 (3)	0.016 (3)	0.002 (3)
C20	0.020 (2)	0.027 (3)	0.013 (2)	−0.0033 (19)	−0.0004 (17)	0.0074 (19)
C7	0.021 (2)	0.029 (3)	0.019 (2)	−0.002 (2)	0.0032 (18)	0.006 (2)
C25	0.025 (3)	0.025 (3)	0.021 (2)	−0.001 (2)	−0.0016 (19)	−0.003 (2)
C23	0.021 (3)	0.037 (3)	0.039 (3)	−0.004 (2)	0.001 (2)	0.011 (3)
C24	0.024 (3)	0.033 (3)	0.029 (3)	−0.001 (2)	0.003 (2)	0.007 (2)
C22	0.031 (3)	0.043 (4)	0.022 (3)	−0.002 (2)	−0.007 (2)	0.005 (2)
C12	0.041 (3)	0.052 (4)	0.027 (3)	0.021 (3)	0.016 (2)	0.012 (3)
C10	0.071 (5)	0.027 (4)	0.024 (3)	0.001 (3)	0.011 (3)	−0.003 (2)
C21	0.022 (3)	0.042 (3)	0.020 (2)	0.002 (2)	−0.0008 (19)	0.005 (2)

### (4-Bromobenzyl)triphenylphosphonium triiodide (III) Geometric parameters (Å, º)

**Table:**

I2—I1	3.0099 (5)	C13—C12	1.398 (9)
I2—I3	2.8603 (5)	C9—H9	0.9500
Br1—C5	1.886 (5)	C9—C10	1.384 (8)
P1—C8	1.790 (5)	C17—H17	0.9500
P1—C14	1.800 (5)	C17—C16	1.381 (8)
P1—C1	1.818 (4)	C1—H1A	0.9900
P1—C20	1.799 (5)	C1—H1B	0.9900
C8—C13	1.392 (8)	C3—H3	0.9500
C8—C9	1.391 (8)	C16—H16	0.9500
C6—H6	0.9500	C11—H11	0.9500
C6—C5	1.390 (7)	C11—C12	1.371 (11)
C6—C7	1.377 (8)	C11—C10	1.388 (10)
C5—C4	1.389 (8)	C20—C25	1.382 (7)
C18—H18	0.9500	C20—C21	1.399 (7)
C18—C19	1.387 (8)	C7—H7	0.9500
C18—C17	1.380 (8)	C25—H25	0.9500
C19—H19	0.9500	C25—C24	1.380 (7)
C19—C14	1.388 (7)	C23—H23	0.9500
C14—C15	1.387 (7)	C23—C24	1.385 (8)
C2—C1	1.505 (7)	C23—C22	1.391 (8)
C2—C3	1.386 (7)	C24—H24	0.9500
C2—C7	1.391 (7)	C22—H22	0.9500
C4—H4	0.9500	C22—C21	1.394 (8)
C4—C3	1.381 (8)	C12—H12	0.9500
C15—H15	0.9500	C10—H10	0.9500
C15—C16	1.396 (8)	C21—H21	0.9500
C13—H13	0.9500		
			
I3—I2—I1	173.489 (15)	P1—C1—H1A	109.0
C8—P1—C14	109.4 (2)	P1—C1—H1B	109.0
C8—P1—C1	109.8 (2)	C2—C1—P1	113.1 (3)
C8—P1—C20	109.6 (2)	C2—C1—H1A	109.0
C14—P1—C1	109.1 (2)	C2—C1—H1B	109.0
C20—P1—C14	109.2 (2)	H1A—C1—H1B	107.8
C20—P1—C1	109.8 (2)	C2—C3—H3	119.6
C13—C8—P1	120.2 (4)	C4—C3—C2	120.8 (5)
C9—C8—P1	119.2 (4)	C4—C3—H3	119.6
C9—C8—C13	120.6 (5)	C15—C16—H16	120.0
C5—C6—H6	120.0	C17—C16—C15	120.1 (5)
C7—C6—H6	120.0	C17—C16—H16	120.0
C7—C6—C5	120.0 (5)	C12—C11—H11	119.3
C6—C5—Br1	120.0 (4)	C12—C11—C10	121.3 (6)
C4—C5—Br1	120.5 (4)	C10—C11—H11	119.3
C4—C5—C6	119.5 (5)	C25—C20—P1	120.1 (4)
C19—C18—H18	120.4	C25—C20—C21	119.5 (5)
C17—C18—H18	120.4	C21—C20—P1	120.1 (4)
C17—C18—C19	119.1 (5)	C6—C7—C2	120.9 (5)
C18—C19—H19	119.7	C6—C7—H7	119.5
C18—C19—C14	120.5 (5)	C2—C7—H7	119.5
C14—C19—H19	119.7	C20—C25—H25	119.6
C19—C14—P1	117.7 (4)	C24—C25—C20	120.8 (5)
C15—C14—P1	122.1 (4)	C24—C25—H25	119.6
C15—C14—C19	120.1 (5)	C24—C23—H23	120.0
C3—C2—C1	122.3 (5)	C24—C23—C22	119.9 (5)
C3—C2—C7	118.7 (5)	C22—C23—H23	120.0
C7—C2—C1	119.0 (4)	C25—C24—C23	120.0 (5)
C5—C4—H4	120.0	C25—C24—H24	120.0
C3—C4—C5	120.0 (4)	C23—C24—H24	120.0
C3—C4—H4	120.0	C23—C22—H22	120.0
C14—C15—H15	120.4	C23—C22—C21	120.0 (5)
C14—C15—C16	119.2 (5)	C21—C22—H22	120.0
C16—C15—H15	120.4	C13—C12—H12	120.1
C8—C13—H13	120.5	C11—C12—C13	119.8 (6)
C8—C13—C12	119.0 (6)	C11—C12—H12	120.1
C12—C13—H13	120.5	C9—C10—C11	119.3 (7)
C8—C9—H9	120.1	C9—C10—H10	120.3
C10—C9—C8	119.8 (6)	C11—C10—H10	120.3
C10—C9—H9	120.1	C20—C21—H21	120.2
C18—C17—H17	119.6	C22—C21—C20	119.7 (5)
C18—C17—C16	120.9 (5)	C22—C21—H21	120.2
C16—C17—H17	119.6		
			
Br1—C5—C4—C3	177.2 (4)	C17—C18—C19—C14	−3.1 (9)
P1—C8—C13—C12	−179.1 (4)	C1—P1—C8—C13	−104.2 (4)
P1—C8—C9—C10	179.4 (5)	C1—P1—C8—C9	75.2 (5)
P1—C14—C15—C16	−180.0 (4)	C1—P1—C14—C19	−177.1 (4)
P1—C20—C25—C24	175.5 (4)	C1—P1—C14—C15	1.2 (5)
P1—C20—C21—C22	−174.8 (5)	C1—P1—C20—C25	42.2 (5)
C8—P1—C14—C19	62.8 (5)	C1—P1—C20—C21	−143.5 (5)
C8—P1—C14—C15	−118.9 (5)	C1—C2—C3—C4	−177.5 (4)
C8—P1—C1—C2	−51.8 (4)	C1—C2—C7—C6	176.3 (4)
C8—P1—C20—C25	162.9 (4)	C3—C2—C1—P1	−84.1 (5)
C8—P1—C20—C21	−22.8 (5)	C3—C2—C7—C6	−2.3 (7)
C8—C13—C12—C11	−0.2 (9)	C20—P1—C8—C13	135.1 (4)
C8—C9—C10—C11	−0.3 (10)	C20—P1—C8—C9	−45.5 (5)
C6—C5—C4—C3	−1.4 (7)	C20—P1—C14—C19	−57.2 (5)
C5—C6—C7—C2	1.7 (7)	C20—P1—C14—C15	121.2 (5)
C5—C4—C3—C2	0.7 (7)	C20—P1—C1—C2	68.7 (4)
C18—C19—C14—P1	−178.3 (4)	C20—C25—C24—C23	−1.4 (9)
C18—C19—C14—C15	3.3 (8)	C7—C6—C5—Br1	−178.4 (4)
C18—C17—C16—C15	0.1 (9)	C7—C6—C5—C4	0.1 (7)
C19—C18—C17—C16	1.4 (9)	C7—C2—C1—P1	97.4 (5)
C19—C14—C15—C16	−1.7 (9)	C7—C2—C3—C4	1.1 (7)
C14—P1—C8—C13	15.5 (5)	C25—C20—C21—C22	−0.5 (9)
C14—P1—C8—C9	−165.2 (4)	C23—C22—C21—C20	−0.1 (9)
C14—P1—C1—C2	−171.7 (3)	C24—C23—C22—C21	−0.1 (9)
C14—P1—C20—C25	−77.3 (5)	C22—C23—C24—C25	0.9 (9)
C14—P1—C20—C21	97.0 (5)	C12—C11—C10—C9	1.6 (11)
C14—C15—C16—C17	0.0 (9)	C10—C11—C12—C13	−1.4 (10)
C13—C8—C9—C10	−1.3 (9)	C21—C20—C25—C24	1.2 (8)
C9—C8—C13—C12	1.5 (8)		

### (4-Bromobenzyl)triphenylphosphonium triiodide (III) Hydrogen-bond geometry (Å, º)

**Table:**

*D*—H···*A*	*D*—H	H···*A*	*D*···*A*	*D*—H···*A*
C1—H1*B*···I1^i^	0.99	3.05	3.900 (4)	144
C7—H7···I2	0.95	3.06	3.716 (5)	128

Symmetry code: (i) −*x*+1, −*y*+1, −*z*+1.

## References

[bb1] Chen, X., Chen, W.-Q., Yu, L.-L., Lin, J.-H., Zhou, D.-D., Yin, W.-T., Zuo, H.-R., Zhou, J.-R., Yang, L.-M. & Ni, C.-L. (2011). *J. Mol. Struct.* **1006**, 419–424.

[bb2] Chen, X., Dai, S.-L., Cheng, Z.-P., Liang, L.-B., Han, S., Liu, J.-F., Zhou, J.-R., Yang, L.-M. & Ni, C.-L. (2012). *Synth. React. Inorg. Met.-Org. Nano-Met. Chem.* **42**, 987–993.

[bb3] Chernov’yants, M. S., Burykin, I. V., Starikova, Z. A. & Rassoshenko, A. A. (2016). *Zh. Neorg. Khim.* **61**, 217–220.

[bb4] Dolomanov, O. V., Bourhis, L. J., Gildea, R. J., Howard, J. A. K. & Puschmann, H. (2009). *J. Appl. Cryst.* **42**, 339–341.

[bb5] Fang, S. F., Li, H. N., Xie, Y. I., Li, H. X., Wang, Y. & Shi, Y. M. (2021). *Small* **17**, 2103831. 10.1002/smll.202103831 34546632

[bb6] Gong, Z., Zhang, J., Deng, X., Ren, M.-P., Wang, W.-Q., Wang, Y.-J., Cao, H., Wang, L., He, Y.-C. & Lei, X.-W. (2024). *Aggregate* **5**, e574.

[bb7] Groom, C. R., Bruno, I. J., Lightfoot, M. P. & Ward, S. C. (2016). *Acta Cryst.* B**72**, 171–179. 10.1107/S2052520616003954 PMC4822653 27048719

[bb8] Li, D. Y., Song, J. H., Xu, Z. Y., Gao, Y. J., Yin, X., Hou, Y. H., Feng, L. J., Yue, C. Y., Fei, H. H. & Lei, X. W. (2022). *Chem. Mater.* **34**, 6985–6995.

[bb9] Li, X. & Wang, G. (2025*a*). *Z. Kristallogr. New Cryst. Struct.* **240**, 407–409.

[bb10] Li, X. & Wang, G. (202*b*5). *Z. Kristallogr. New Cryst. Struct.* **240**, 411–412.

[bb11] McKinnon, J. J., Jayatilaka, D. & Spackman, M. A. (2007). *Chem. Commun.* pp. 3814–3816. 10.1039/b704980c 18217656

[bb12] Rigaku OD (2025). *CrysAlis PRO*. Rigaku Oxford Diffraction, Yarnton, England.

[bb13] Sheldrick, G. M. (2015*a*). *Acta Cryst.* A**71**, 3–8.

[bb14] Sheldrick, G. M. (2015*b*). *Acta Cryst.* C**71**, 3–8.

[bb15] Spackman, P. R., Turner, M. J., McKinnon, J. J., Wolff, S. K., Grimwood, D. J., Jayatilaka, D. & Spackman, M. A. (2021). *J. Appl. Cryst.* **54**, 1006–1011. 10.1107/S1600576721002910 PMC8202033 34188619

[bb16] Vogt, H., Wulff-Molder, D. & Meisel, M. (1996). *Z. Naturforsch. B: Chem. Sci.* **51**, 1443–1448.

[bb17] Xu, C., Zhang, J., Pan, Y., Liu, Y., Yang, X., Xiao, H. & Zou, J. (2025). *Materials Today Chemistry* **49**, 102975.

[bb18] Xu, L. J., Lin, X., He, Q., Worku, M. & Ma, B. (2020). *Nat. Commun.* pp. 11 No. 4329. 10.1038/s41467-020-18119-y PMC7455565 32859920

[bb19] Yang, L., Shi, C., Li, B., Liu, X., Gao, X., Li, Y. & Xu, Y. (2025). *Inorg. Chem. Front.* **12**, 1528–1537.

[bb20] Yang, S.-B. & Ni, C.-L. (2006). *Acta Cryst.* E**62**, m483–m485.

[bb21] Yu, Y., Liu, S., Zhang, J., Zhao, W., Tang, Y., Han, C., Chen, X., Xu, L., Chen, R., Li, M., Tao, Y. & Lv, W. (2024). *Inorg. Chem.* **63**, 10296–10303. 10.1021/acs.inorgchem.4c00978 38776123

